# Analysis of early-pregnancy metabolome in early- and late-onset gestational diabetes reveals distinct associations with maternal overweight

**DOI:** 10.1007/s00125-024-06237-x

**Published:** 2024-07-31

**Authors:** Senja Masalin, Anton Klåvus, Kristiina Rönö, Heikki A. Koistinen, Ville Koistinen, Olli Kärkkäinen, Tiina J. Jääskeläinen, Miira M. Klemetti

**Affiliations:** 1 Department of Obstetrics and Gynecology, University of Helsinki and Helsinki University Hospital, Helsinki, Finland; 2 Obstetrics and Gynecology, South Karelia Central Hospital, Lappeenranta, Finland; 3 Department of General Practice and Primary Healthcare, University of Helsinki and Helsinki University Hospital, Helsinki, Finland; 4 Afekta Technologies Ltd, Kuopio, Finland; 5 Department of Medicine, University of Helsinki and Helsinki University Hospital, Helsinki, Finland; 6 Minerva Foundation Institute for Medical Research, Helsinki, Finland; 7 School of Pharmacy, University of Eastern Finland, Kuopio, Finland; 8 Department of Medical and Clinical Genetics, University of Helsinki, Helsinki, Finland; 9 Department of Food and Nutrition, University of Helsinki, Helsinki, Finland

**Keywords:** Early pregnancy, Gestational diabetes, Insulin resistance, LC-MS, Metabolism, OGTT, Overweight, Untargeted metabolomics, Waist circumference

## Abstract

**Aims/hypothesis:**

It is not known whether the early-pregnancy metabolome differs in patients with early- vs late-onset gestational diabetes mellitus (GDM) stratified by maternal overweight. The aims of this study were to analyse correlations between early-pregnancy metabolites and maternal glycaemic and anthropometric characteristics, and to identify early-pregnancy metabolomic alterations that characterise lean women (BMI <25 kg/m^2^) and women with overweight (BMI ≥25 kg/m^2^) with early-onset GDM (E-GDM) or late-onset GDM (L-GDM).

**Methods:**

We performed a nested case–control study within the population-based prospective Early Diagnosis of Diabetes in Pregnancy cohort, comprising 210 participants with GDM (126 early-onset, 84 late-onset) and 209 normoglycaemic control participants matched according to maternal age, BMI class and primiparity. Maternal weight, height and waist circumference were measured at 8–14 weeks’ gestation. A 2 h 75 g OGTT was performed at 12–16 weeks’ gestation (OGTT1), and women with normal results underwent repeat testing at 24–28 weeks’ gestation (OGTT2). Comprehensive metabolomic profiling of fasting serum samples, collected at OGTT1, was performed by untargeted ultra-HPLC-MS. Linear models were applied to study correlations between early-pregnancy metabolites and maternal glucose concentrations during OGTT1, fasting insulin, HOMA-IR, BMI and waist circumference. Early-pregnancy metabolomic features for GDM subtypes (participants stratified by maternal overweight and gestational timepoint at GDM onset) were studied using linear and multivariate models. The false discovery rate was controlled using the Benjamini–Hochberg method.

**Results:**

In the total cohort (*n*=419), the clearest correlation patterns were observed between (1) maternal glucose concentrations and long-chain fatty acids and medium- and long-chain acylcarnitines; (2) maternal BMI and/or waist circumference and long-chain fatty acids, medium- and long-chain acylcarnitines, phospholipids, and aromatic and branched-chain amino acids; and (3) HOMA-IR and/or fasting insulin and l-tyrosine, certain long-chain fatty acids and phospholipids (*q*<0.001). Univariate analyses of GDM subtypes revealed significant differences (*q*<0.05) for seven non-glucose metabolites only in overweight women with E-GDM compared with control participants: linolenic acid, oleic acid, docosapentaenoic acid, docosatetraenoic acid and lysophosphatidylcholine 20:4/0:0 abundances were higher, whereas levels of specific phosphatidylcholines (P-16:0/18:2 and 15:0/18:2) were lower. However, multivariate analyses exploring the early-pregnancy metabolome of GDM subtypes showed differential clustering of acylcarnitines and long-chain fatty acids between normal-weight and overweight women with E- and L-GDM.

**Conclusions/interpretation:**

GDM subtypes show distinct early-pregnancy metabolomic features that correlate with maternal glycaemic and anthropometric characteristics. The patterns identified suggest early-pregnancy disturbances of maternal lipid metabolism, with most alterations observed in overweight women with E-GDM. Our findings highlight the importance of maternal adiposity as the primary target for prevention and treatment.

**Graphical Abstract:**

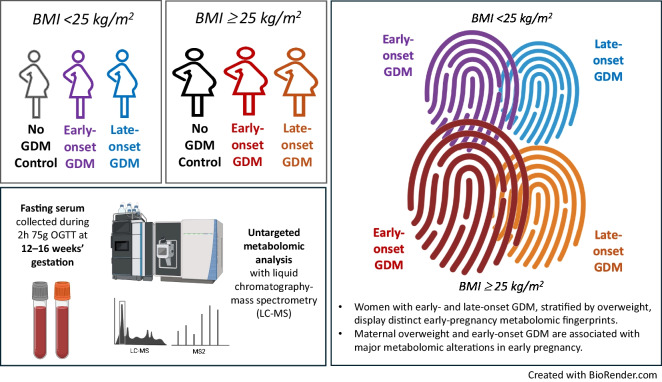

**Supplementary Information:**

The online version contains peer-reviewed but unedited supplementary material available at 10.1007/s00125-024-06237-x.


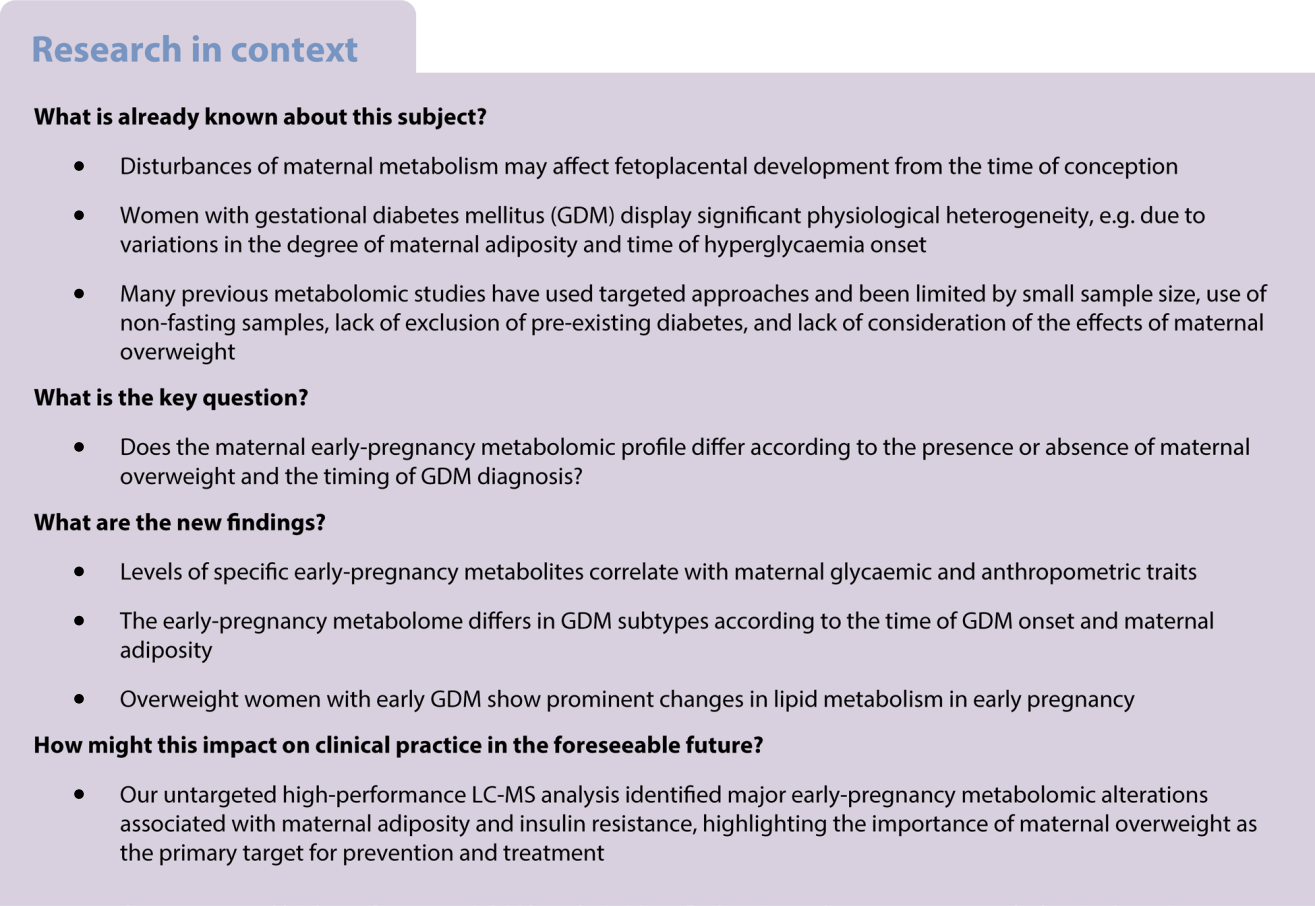


## Introduction

Gestational diabetes mellitus (GDM) is defined as hyperglycaemia detected for the first time during pregnancy [1], excluding overt diabetes. Increasing frequencies of up to 15% are seen internationally, paralleling the obesity epidemic [1, 2]. Both GDM and maternal obesity are associated with adverse short- and long-term health outcomes in the mothers and their offspring [1].

Recent evidence suggests that women with GDM display heterogeneity in terms of both pathophysiology and clinical outcomes [3, 4]. The dominant defect may be excess insulin resistance, insufficient insulin secretion, or both [5]. Other sources of heterogeneity are the presence or absence of maternal overweight [6] and the gestational timepoint at GDM onset [7]. Although, GDM is traditionally diagnosed in the latter half of gestation, increasing recognition is being given to the maternal metabolic environment as a central determinant of fetoplacental development from the time of conception [8]. Interestingly, a recent RCT showed that early treatment of women with GDM diagnosed at <20 weeks’ gestation improves neonatal outcomes [9].

Studies on type 2 diabetes, obesity and insulin resistance have revealed various typical metabolomic signatures [10, 11]. However, findings from metabolomic studies on GDM have been inconsistent [12, 13], possibly due to small sample sizes and lack of consideration of the confounding effects of maternal overweight [14–16] or other phenotypic differences between GDM subtypes [17]. Further, many studies collected maternal samples only in the second half of pregnancy, preventing examination of critical early-pregnancy metabolism. A recent study showed unique metabolomic and genetic patterns in insulin-deficient and insulin-resistant GDM subtypes in late pregnancy [18]. Whether the early-pregnancy metabolome differs in lean and overweight GDM subtypes, or depends on the gestational timepoint at GDM onset, remains unclear [7, 19].

The present study is a nested case–control study within the Finnish prospective, population-based Early Diagnosis of Diabetes in Pregnancy (EDDIE) cohort [20]. Using untargeted ultra-HPLC-MS, we aimed to analyse correlations between early-pregnancy metabolites and maternal glycaemic and anthropometric characteristics (fasting and post-load glucose concentrations, fasting insulin, HOMA-IR, maternal BMI and waist circumference [WC]) and to identify early-pregnancy metabolomic alterations that characterise normal-weight and overweight women with early-onset GDM (E-GDM) diagnosed at 12–16 weeks’ gestation or late-onset GDM (L-GDM) diagnosed at 24–28 weeks’ gestation.

## Methods

### Study participants and sample collection

The EDDIE cohort is a prospective, population-based cohort of 1605 pregnant women from South Karelia, Finland, between 2013 and 2016. Details of cohort formation have been published previously [20]. Briefly, 2305 pregnant women were asked to participate in the study during the first ultrasound screening appointment at South Karelia Central Hospital (Lappeenranta, Finland) or Honkaharju Hospital (Imatra, Finland) between 8 and 14 weeks’ gestation. Of these women, 527 (22.9%) declined to participate and 173 (7.5%) were excluded. Exclusion criteria were previously diagnosed type 1 or type 2 diabetes, use of a medication that affects glucose metabolism, such as corticosteroids, or the inability to understand the consent forms due to insufficient language skills. Although data on ethnicity was not collected, the Finnish population is relatively homogenous in terms of ethnicity, with most being of European descent.

Maternal height, weight and WC were measured, venous blood samples were drawn for HbA_1c_ analysis, and information about pregnancy history and diabetes risk factors was collected using a structured questionnaire. All participants were referred for a 2 h 75 g OGTT between 12 and 16 weeks’ gestation (OGTT1). Additional fasting serum samples were collected at the time of the OGTT1.

GDM was diagnosed according to the Finnish Current Care Guidelines [21] if any of the following thresholds were met or exceeded: 0 h (fasting plasma) glucose ≥5.3 mmol/l, 1 h glucose ≥10.0 mmol/ or 2 h glucose ≥8.6 mmol/l. Participants who had a normal OGTT1 were referred for a repeat OGTT at 24–28 weeks’ gestation (OGTT2). The same diagnostic thresholds were used to diagnose E-GDM at OGTT1 and L-GDM at OGTT2 [21].

For the present case–control study, we initially selected all EDDIE participants with E-GDM or L-GDM for whom a fasting serum sample collected at OGTT1 was available. Women who reported smoking during pregnancy and those with thyroid, autoimmune or hypertensive disorders requiring medication were excluded due to potential effects on the early-pregnancy metabolome. In total, we identified 210 women with GDM who met the inclusion criteria: 126 women with E-GDM (abnormal OGTT1) and 84 women with L-GDM (normal OGTT1, abnormal OGTT2). Forty women were treated with insulin and/or metformin (29 women with E-GDM and 11 women with L-GDM). These women were matched with 210 control women (both OGTT1 and OGTT2 normal) according to age (±3 years), primiparity (yes/no) and BMI class (<18.5 kg/m^2^, 18.5–24.99 kg/m^2^, 25–29.99 kg/m^2^, 30–35.99 kg/m^2^, ≥40 kg/m^2^). The case–control matching function in SPSS Statistics 26.0 for Mac (IBM, USA) was used for matching of GDM patients and control participants.

### Analysis of HbA_1c_, plasma glucose and serum insulin concentrations

HbA1_c_ and plasma glucose concentrations during the OGTTs were analysed at the South Karelia Central Hospital Laboratory from fresh venous blood samples. Fasting serum insulin was analysed at Vita Laboratories (Helsinki, Finland) from serum samples collected at the time of OGTT1 and frozen at −80°C until analysis (see electronic supplementary material [ESM] Methods for further details).

### Sample preparation and LC-MS analysis

Fasting serum samples, stored at −80°C until use, were analysed by Afekta Technologies (Kuopio, Finland) using LC-MS, comprising a Vanquish Flex UHPLC system (Thermo Scientific, Germany) coupled with a high-resolution Orbitrap mass spectrometer (Q Exactive Focus, Thermo Scientific). Sample preparation and the analytical methods are described in detail in the ESM Methods and in previous publications [22, 23]. In brief, a Zorbax Eclipse XDB-C18 column (2.1×100 mm, particle size 1.8 µm; Agilent Technologies, USA) was used for the reversed-phase separation, and an Aqcuity UPLC BEH amide column (Waters, USA) was used for the hydrophilic interaction liquid chromatography separation, combined with jet stream electrospray ionisation in both positive and negative modes. The samples were analysed in three batches comprising 154, 165 and 165 samples, including quality control samples. The same quality control samples were used in all batches to enable batch effect correction. One control sample was inadvertently omitted from the LC-MS analysis, resulting in a final sample of 209 control participants.

### Data analysis

#### Analysis of clinical data

An unpaired, independent-sample Student’s *t* test, χ^2^ test and ANOVA were used to compare characteristics between the groups. In cases of violation of the normality assumptions, a permutation *t* test or a bootstrap-type method was applied. The significance of pairwise multiple comparisons was corrected using the Šídák method. The normality of variables was tested using the Shapiro–Wilk test. Insulin resistance was quantified using HOMA-IR [24]. The Stata 17 statistical package (StataCorp, USA) was used for the analyses.

#### Analysis of MS data

Peak detection and alignment were performed using MS-DIAL version 4.60 [25]. Minimum peak height was set at 250,000. The peaks were detected using the linear weighted moving-average algorithm. Peak alignment parameters included a retention time tolerance of 0.05 min and an *m*/*z* tolerance of 0.015 Da, with a requirement that the maximum signal be at least five times the mean of the solvent blank samples. Data preprocessing and clean-up steps included drift correction, low-quality feature flagging, imputation and batch effect correction using the notame package, version 0.06 [23]. Batch effects between the three batches were corrected using the batchCorr R package [26].

For univariate analyses, we used linear regression models fitted separately for each molecular feature. The feature levels (peak areas based on signal counts) were used as the dependent variable in all models. Models were run using OGTT1 plasma glucose concentrations, continuous BMI values, fasting insulin concentrations and WC as predictors. Additional models were run to evaluate the effects of GDM groups (control participants, E-GDM, L-GDM and total GDM groups) in the BMI categories (BMI <25 kg/m^2^ vs BMI ≥25 kg/m^2^). In multivariate analyses, a partial least-squares discriminant analysis (PLS-DA) model was applied in the MUVR R package (version 0.0.974, Chalmers University of Technology, Sweden) to identify metabolites that predict group differences. The statistical models are explained in detail in ESM Methods. The Benjamini–Hochberg method was used to control for the false discovery rate. Corrected *p* values (*q* values) <0.05 were considered significant. All metabolomic data analyses were performed using R version 3.6.3 (R Foundation, Austria) and notame version 0.0.6 [23].

### Compound identification

The chromatographic and mass spectrometric characteristics (retention time, exact mass and MS/MS spectra) of the significantly different molecular features were compared with entries in an in-house standard library, publicly available databases and published literature. Annotation of each metabolite and the level of identification were made based on the recommendations of the Chemical Analysis Working Group Metabolomics Standards Initiative [27].

### Ethical approval

All study participants provided written informed consent for the study. The study protocol was approved by the ethical committee of the Hospital District of Helsinki and Uusimaa (343/13/03/03/2012), with the latest amendment accepted on 13 November 2019 (HUS/1794/2016).

## Results

### Maternal and offspring characteristics in the total cohort

Maternal characteristics at the time of enrolment and basic offspring characteristics are summarised in Table 1. GDM and control participants were well-matched for age, maternal BMI and primiparity. Birthweight, fetal sex distribution and gestational age at birth were similar between the groups. In contrast, women with GDM had larger WC and higher BP, glucose concentrations at OGTT1, HbA_1c_ levels, fasting insulin concentrations and HOMA-IR (all *p* values <0.05).

**Table 1:** Maternal and basic offspring characteristics of women with GDM vs normoglycaemic control participants, selected from the Finnish Early Diagnosis of Diabetes in Pregnancy cohort and matched according to age, primiparity and BMI class

	GDM (*n*=210)	Control (*n*=209)	*p* value
Maternal characteristics			
Age (years)	31.4 ± 4.9	31.2 ± 4.5	0.69
BMI (kg/m^2^)	27.8 ± 5.3	27.3 ± 5.1	0.30
BMI class (kg/m^2^)			NA
Normal (18.5–24.99)	76 (36)	76 (36)	
Overweight (25–29.99)	74 (35)	74 (35)	
Obesity class I (30–34.99)	40 (19)	40 (19)	
Obesity class II (35–39.99)	15 (7)	15 (7)	
Obesity class III (≥40)	5 (2)	4 (2)	
Weight (kg)	76.0 ± 14.8	75.1 ± 15.6	0.58
WC (cm)	89.8 ± 10.7 [209]	87.3 ± 10.2 [202]	0.02
Systolic BP (mmHg)	120 ± 10	117 ± 10 [206]	0.02
Diastolic BP (mmHg)	77 ± 8	75 ± 9 [206]	0.046
OGTT1			
Gestational age (weeks)	14.3 ± 0.9	14.5 ± 0.9	0.01
Fasting glucose (mmol/l)	5.1 ± 0.4	4.8 ± 0.2	<0.001
1 h post-load glucose (mmol/l)	8.0 ± 1.9	6.3 ± 1.3	<0.001
2 h post-load glucose (mmol/l)	6.7 ± 1.4	5.3 ± 1.0	<0.001
E-GDM	126 (60)	–	–
HbA_1c_ (mmol/mol)	34.8 ± 3.4 [209]	33.1 ± 2.9 [208]	<0.001
HbA_1c_ (%)	5.3 ± 2.5	5.2 ± 2.4	<0.001
Fasting serum insulin (pmol/l)	74.31 ± 52.78 [192]	55.56 ± 27.09 [195]	<0.001
HOMA-IR	2.46 ± 1.69 [192]	1.71 ± 0.87 [195]	<0.001
Primiparous	89 (42)	90 (43)	0.89
Prior GDM (multiparas only)	44 (36) [121]	8 (7) [119]	<0.001
Family history of diabetes^a^	100 (48)	90 (43)	0.35
Hypertension during pregnancy^b^	29 (14)	27 (13)	0.79
Offspring characteristics			
Birthweight (g)	3625 ± 528	3549 ± 566	0.15
Male	102 (49)	114 (55)	0.22
Gestational age at birth (weeks)	39.7 ± 1.8	39.9 ± 1.9	0.25

Data are means ± SD for continuous variables and *n* (%) for categorical variables. If data were only available for a subset of participants or only applicable to a subset of participants, the number of participants is included in square brackets

^a^Self-reported data

^b^Hypertensive participants were non-medicated and not diagnosed before pregnancy; diagnoses of pre-eclampsia were excluded

NA, not applicable

### Overlapping and distinct early-pregnancy metabolomic alterations are observed in GDM and maternal overweight

Overall, 7259 good-quality molecular features were included in the statistical analyses after data preprocessing and clean-up. Initially we examined the entire cohort of 419 women to obtain an overview of the early-pregnancy metabolites that differ between GDM/non-GDM participants and overweight or normal-weight participants.

#### GDM vs control women

**Fig. 1:**
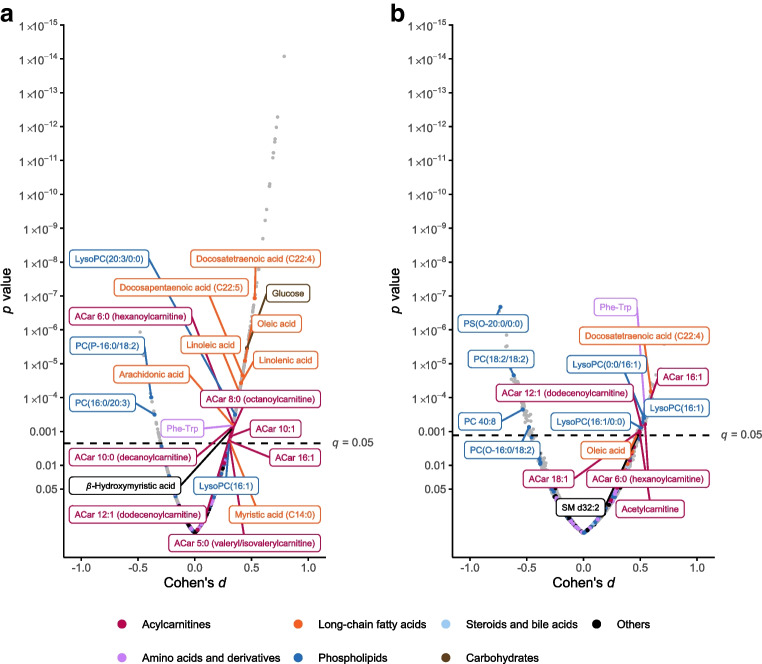
Volcano plots showing differential metabolites associated with (**a**) GDM (regardless of BMI and time of GDM onset) compared with matched control participants without GDM and (**b**) maternal overweight in women without GDM compared with normal-weight control participants without GDM. Identified metabolites with statistically significant alterations in abundance are labelled by colour according to group: long-chain fatty acids (orange), acylcarnitines (red), derivatives of phospholipids (blue), carbohydrates (brown) and amino acids (purple). Grey dots indicate unidentified metabolites. The dashed line indicates level of significance (*q* value <0.05). PS, phosphatidylserine; SM, sphingomyelin

First, we compared all women diagnosed with GDM, regardless of BMI and time of GDM onset, with matched control participants (Fig. 1a). Several long-chain fatty acids, medium- and long-chain acylcarnitines (ACar), a few lysophosphatidylcholines (lysoPCs) and phenylalanyltryptophan (Phe-Trp, a dipeptide formed from l-phenylalanine and l-tryptophan residues) were identified as present in higher abundance in GDM women compared with control participants (*q*<0.05). In contrast, a few phosphatidylcholine (PC) species were present in lower abundance.

#### Overweight vs normal-weight control participants

Next we compared overweight and normal-weight control participants without GDM (Fig. 1b). The abundances of long-chain fatty acids, e.g. docosatetraenoic acid (FA C22:4) and oleic acid, medium- and long-chain ACar, lysoPCs, Phe-Trp and sphingomyelin d32:2 were increased, and that of certain PCs and phosphatidylserine O-20:0/0:0 was decreased, in overweight control participants vs normal-weight control participants (*q*<0.05).

### Maternal glycaemic and anthropometric traits correlate with early-pregnancy lipid, acylcarnitine and amino acid metabolites

**Fig. 2:**
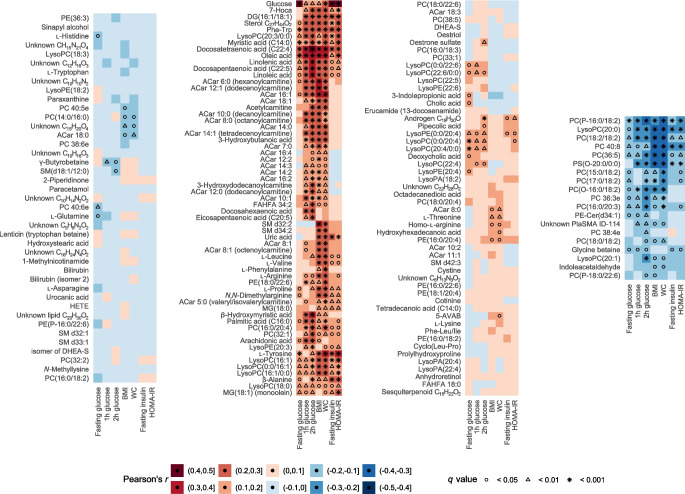
Heatmaps illustrating the clustering and correlations of annotated early-pregnancy metabolites with maternal plasma glucose concentrations (fasting, 1 h and 2 h post-load plasma glucose during an OGTT at 12–16 weeks’ gestation on a continuous scale), maternal BMI and WC, and maternal fasting serum insulin and HOMA-IR. The level of significance is indicated with differently shaped symbols (circles, triangles, stars) . 5-AVAB, 5-Aminovaleric acid betaine; DG, diacylglycerol; DHEA-S, dehydroepiandrosterone sulfate; FAHFA, fatty acid ester of hydroxy fatty acid; HETE, hydroxyeicosatetraenoic acid; 7-HOCA, 7α-hydroxy-3-oxo-4-cholestenoic acid; LysoPE, lysophosphatidyl ethanolamine; MG, monoglyceride; PE, phosphatidyl ethanolamine PS, phosphatidylserine; SM, sphingomyelin

As overlapping metabolomic alterations were detected in GDM and maternal overweight, we continued our analyses by exploring correlations between maternal glycaemic and anthropometric variables and early-pregnancy metabolites. The heatmap in Fig. 2 demonstrates the direction, strength and statistical significance of Pearson correlations between detected metabolites and (1) glucose concentrations during OGTT1 at 12–16 weeks’ gestation; (2) maternal BMI and WC; and (3) fasting insulin concentrations and HOMA-IR, in the entire cohort. We observed the correlations described below, with a correlation coefficient greater than 0.2 and a *q* value <0.001.

#### Fasting glucose concentrations

Fasting plasma glucose at OGTT1 correlated moderately only with a few metabolites, e.g. docosatetraenoic acid (FA C22:4) and oleic acid.

#### Post-load glucose concentrations

Glucose concentrations 1 h and 2 h post-load correlated most strongly with docosatetraenoic acid and oleic acid, and these correlations were stronger than those observed for fasting plasma glucose. Post-load glucose concentrations, particularly the 2 h glucose value, also correlated with other long-chain fatty acids. Weaker but statistically significant correlations were found between 1 h and/or 2 h post-load glucose concentrations and medium and long-chain ACar, Phe-Trp, certain cholesterol metabolites (7α-hydroxy-3-oxo-4-cholestenoic acid and 7-ketocholesterol [sterol C_27_H_44_O_22_]), diacylglycerol 16:1/18:1, and specific phospholipids (PC 16:0/20:4, PC 32:1 and lysoPC 20:3/0:0).

#### Maternal BMI and WC

BMI and WC correlated with long-chain fatty acids and ACar, particularly with docosatetraenoic acid, oleic acid, and ACar 6:0, 12:1 and 16:1. Sphingomyelin d32:2 showed a stronger correlation with maternal BMI, whereas the aromatic amino acids (AAA) l-tyrosine and phenylalanine correlated most strongly with maternal WC. In addition, BMI and WC correlated positively with branched-chain amino acids (BCAA) and other amino acids, Phe-Trp, certain cholesterol metabolites and lysoPCs 16:1, 16:1/0:0, 0:0/16:1 and 20:3/0:0, and negatively with certain PCs, lysoPC 20:0 and phosphatidylserine O-20:0/0:0.

#### Fasting insulin and HOMA-IR

HOMA-IR correlated most strongly with l-tyrosine. A weaker correlation was observed between fasting insulin and l-tyrosine. Weaker correlations were also seen between both parameters of insulin resistance and β-alanine, Phe-Trp, diacylglycerol 16:1/18:1 and docosatetraenoic acid. HOMA-IR also correlated with myristic acid, oleic acid, lysoPCs 20:3/0:0 and 16:1. LysoPC 20:0 and PC 40:8 correlated negatively with both HOMA-IR and fasting insulin.

### Maternal and offspring characteristics in GDM subtypes

For the analysis of the early-pregnancy metabolome in GDM subtypes, we stratified our study participants by the time of GDM onset and maternal overweight. Table 2 compares maternal background characteristics in normal-weight and overweight E-GDM, L-GDM and control women.

**Table 2:** Maternal and basic offspring characteristics for normal-weight and overweight/obese women with E-GDM, L-GDM or no GDM (control participants) selected from the Finnish Early Diagnosis of Diabetes in Pregnancy cohort

	BMI <25 kg/m^2^			*p* value^a^	BMI ≥25 kg/m^2^			*p* value^a^
	E-GDM (A) (*n*=39)	L-GDM (B) (*n*=37)	Control (C) (*n*=76)		E-GDM (D) (*n*=87)	L-GDM (E) (*n*=47)	Control (F) (*n*=133)	
Maternal characteristics								
Age (years)	30.8 ± 5.2	31.3 ± 5.4	31.1 ± 5.0	0.94	32.0 ± 5.1	30.7 ± 4.1	31.2 ± 4.3	0.28
BMI (kg/m^2^)	22.7 ± 1.6	23.3 ± 1.2	22.3 ± 1.6	<0.001 (BC)	30.4 ± 4.8	30.7 ± 4.8	30.2 ± 4.0	0.78
BMI class (kg/m^2^)				NA				0.93
Normal (18.5–24.99)	39 (100)	37 (100)	76 (100)		–	–	–	
Overweight (25–24.99)	–	–	–		50 (57)	24 (51)	74 (56)	
Obesity class I (25–29.99)	–	–	–		25 (29)	15 (32)	40 (30)	
Obesity class II (30–34.99)	–	–	–		8 (9)	7 (15)	15 (11)	
Obesity class III (≥40)	–	–	–		4 (5)	1 (2)	4 (3)	
Weight (kg)	62.9 ± 4.8	63.0 ± 5.5	61.4 ± 6.2	0.24	82.7 ± 13.7	84.4 ± 13.0	82.9 ± 13.9	0.76
WC (cm)	80.0 ± 5.2	80.9 ± 4.8	78.3 ± 5.1 [73]	0.02 (BC)	95.4 ± 9.4	94.5 ± 9.5	92.3 ± 8.9 [129]	0.043 (DF)
Systolic BP (mmHg)	118 ± 11	121 ± 11	115 ± 10 [74]	0.02 (BC)	119 ± 9	120 ± 9	119 ± 10 [132]	0.78
Diastolic BP (mmHg)	74 ± 8	77 ± 6	72 ± 9 [74]	0.005 (BC)	78 ± 8	77 ± 6	77 ± 9 [132]	0.54
OGTT1								
Gestational age (weeks)	14.3 ± 1.0	14.4 ± 1.1	14.7 ± 0.9	0.10	14.2 ± 0.9	14.3 ± 0.9	14.4 ± 0.9	0.23
Fasting glucose (mmol/l)	5.2 ± 0.4	4.9 ± 0.3	4.7 ± 0.2	<0.001 (AB, AC, BC)	5.3 ± 0.3	4.9 ± 0.2	4.8 ± 0.2	<0.001 (DE, DF, EF)
1 h post-load glucose (mmol/l)	8.3 ± 2.2	7.3 ± 1.5	6.1 ± 1.4	<0.001 (AC, BC)	8.5 ± 2.0	7.5 ± 1.4	6.4 ± 1.2	<0.001 (DE, DF, EF)
2 h post-load glucose (mmol/l)	6.5 ± 1.5	6.0 ± 1.1	5.2 ± 1.0	<0.001 (AC, BC)	7.2 ± 1.5	6.4 ± 1.1	5.4 ± 1.0	<0.001 (DE, DF, EF)
HbA_1c_ (mmol/mol)	35.0 ± 3.0	33.4 ± 3.3	33.0 ± 3.1	0.005 (AC)	35.2 ± 3.8 [86]	35.0 ± 2.7	33.1 ± 2.9 [132]	<0.001 (DF, EF)
HbA_1c_ (%)	5.4 ± 2.4	5.2 ± 2.5	5.2 ± 2.4	0.005 (AC)	5.4 ± 2.5 [86]	5.4 ± 2.4	5.2 ± 2.4 [132]	<0.001 (DF, EF)
Fasting serum insulin (pmol/l)	56.95 ± 36.81 [37]	50.00 ± 20.84 [33]	40.28 ± 15.97 [73]	0.003 (AC, BC)	91.67 ± 65.28 [77]	77.78 ± 44.45 [45]	64.59 ± 29.17 [122]	<0.001 (DF)
HOMA-IR	1.91 ± 1.12 [37]	1.55 ± 0.65 [33]	1.23 ± 0.51 [73]	<0.001 (AC, AB)	3.11 ± 2.08 [77]	2.46 ± 1.42 [45]	1.99 ± 0.92 [122]	<0.001 (DF)
Primiparous	16 (41)	18 (49)	35 (46)	0.79	41 (47)	14 (30)	55 (41)	0.15
Prior GDM (multiparas only)	9 (39) [23]	3 (16) [19]	1 (2) [41]	0.001 (AC)	24 (52) [46]	8 (24) [33]	7 (9) [78]	<0.001
Family history of diabetes^b^	22 (56)	13 (35)	29 (38)	0.11	43 (49)	22 (47)	61 (46)	0.87
Hypertension during pregnancy^c^	5 (13)	4 (11)	10 (13)	0.94	13 (15)	7 (15)	17 (13)	0.88
Offspring characteristics								
Birthweight (g)	3520 ± 488	3521 ± 533	3383 ± 617	0.34	3626 ± 583	3794 ± 405	3643 ± 513	0.08
Male	22 (56)	20 (54)	44 (58)	0.93	39 (45)	21 (45)	70 (53)	0.44
Gestational age at birth (weeks)	39.5 ± 1.3	39.9 ± 1.4	39.4 ± 2.2	0.22	39.6 ± 2.3	40.0 ± 1.2	40.3 ± 1.5	0.053

Data are means ± SD for continuous variables and *n* (%) for categorical variables. If data were only available for a subset of participants or only applicable to a subset of participants, the number of participants is included in square brackets

^a^The letters after the *p* values indicate the pairwise comparison between groups to which the value applies. The column headings indicate the letter that applies to each group

^b^Self-reported data

^c^Hypertensive participants were non-medicated and not diagnosed before pregnancy; diagnoses of pre-eclampsia were excluded

*p* values are for pairwise comparisons corrected using the Šídák method

NA, not applicable

#### BMI <25 kg/m^2^

Normal-weight women with GDM had higher glucose concentrations at all time points of OGTT1 (fasting, 1 h and 2 h glucose) and higher fasting insulin concentrations than control participants (all *p*<0.05). Those with E-GDM had higher HOMA-IR compared with L-GDM and control participants (both *p*<0.05). Women with L-GDM had higher early-pregnancy BP and larger WCs compared with normal-weight control participants (both *p*<0.05). Basic offspring characteristics did not differ between the groups.

#### BMI ≥25 kg/m^2^

As expected, overweight women with E-GDM had higher glucose concentrations at all time points of OGTT1 than overweight L-GDM and control women (all *p*<0.001). HbA_1c_ levels were also higher in overweight women with GDM vs control participants (*p*<0.001). Differences in fasting insulin, maternal WC and HOMA-IR were observed only between overweight E-GDM women and control participants (all *p*<0.001). Maternal early-pregnancy BP and basic offspring characteristics did not differ between the groups.

### Alterations in early-pregnancy lipid metabolism characterise overweight women with gestational diabetes

**Fig. 3:**
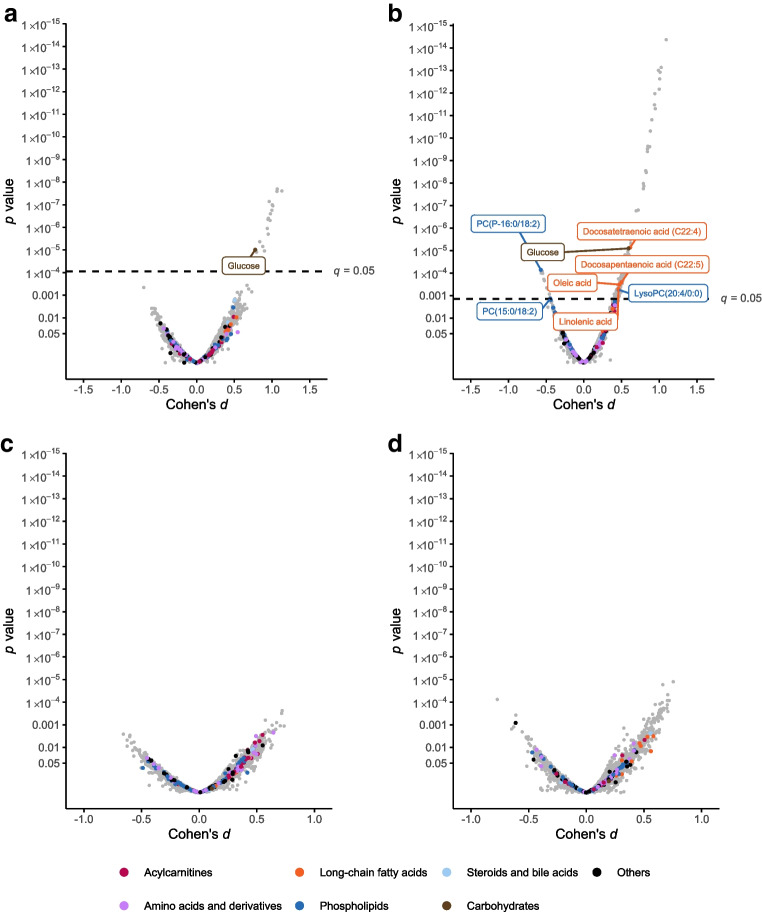
Volcano plots showing differential metabolites associated with (**a**) E-GDM in normal-weight individuals (BMI <25 kg/m^2^) vs normal-weight control participants, (**b**) E-GDM in overweight individuals (BMI ≥25 kg/m^2^) vs overweight control participants, (**c**) L-GDM in normal-weight individuals vs normal-weight control participants, and (**d**) L-GDM in overweight individuals vs overweight control participants. Identified metabolites are labelled by colour according to main metabolite group: long-chain fatty acids (orange), acylcarnitines (red), derivatives of phospholipids (blue), carbohydrates (brown) and amino acids (purple) and those with statistically significant alterations in abundance are named. Grey dots indicate unidentified metabolites. The dashed line in (**a**) and (**b**) indicates the level of significance (*q*<0.05)

Univariate linear models revealed 162 identified or putatively annotated metabolites that had differential mean abundances between normal-weight and overweight women with E-GDM or L-GDM compared with control participants (raw *p* value <0.05) (ESM Table 1). Eight metabolites reached a *q* value <0.05 (glucose, lysoPC 20:4/0:0, lysoPC 20:3/0:0, PC P–16:0/18:2, PC 15:0/18:2, PC 16:0/20:3, linolenic acid, linoleic acid, oleic acid, docosapentaenoic acid [FA C22:5] and docosatetraenoic acid [FA C22:4]). In volcano plot analyses comparing early-pregnancy metabolites in women with E-GDM and L-GDM stratified by BMI, glucose was present in higher abundance in women with E-GDM regardless of BMI (Fig. 3a,b). All significantly differing non-glucose metabolites were detected in overweight women with E-GDM (Fig. 3b). Most metabolites were detected in higher abundance, although metabolites with lower abundances were also observed (e.g. PC P-16:0/18:2 and PC 15:0/18:2). Volcano plot analyses did not reveal statistically significant differences in the early-pregnancy metabolome of normal-weight women with L-GDM vs normal-weight control participants (Fig. 3c), or between overweight women with L-GDM vs overweight control participants (Fig. 3d).

**Fig. 4:**
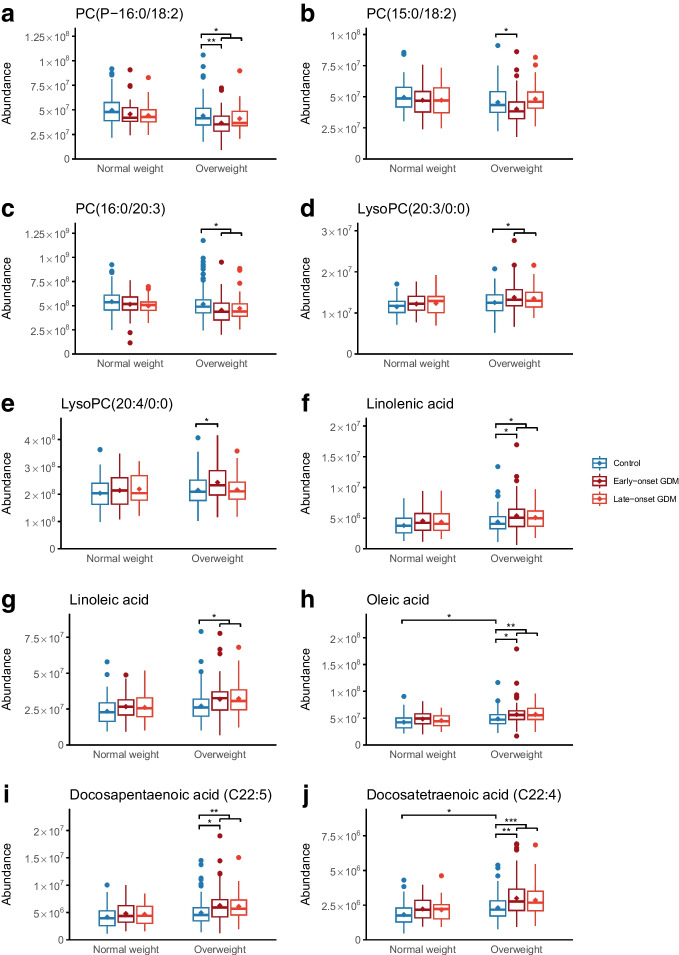
Box plots of significantly differential PCs, lysoPCs and long-chain fatty acids in comparisons of E-GDM and L-GDM vs control participants for normal-weight individuals (BMI <25 kg/m^2^) and overweight individuals (BMI ≥25 kg/m^2^). E-GDM, normal weight: *n*=39; L-GDM, normal weight: *n*=37; control participants, normal weight: *n*=76; E-GDM, overweight: *n*=87; L-GDM, overweight: *n*=47; control participants, overweight: *n*=134. Brackets indicate statistically significant differences between two groups; combined brackets indicate statistically significant differences between a combined group and control participants. Asterisks indicate significant differences for the comparisons between the subgroups: *q<0.05, **q<0.01, ***q<0.001

The box plots in Fig. 4 show metabolites with statistically significant differential abundance in either E-GDM or L-GDM vs control participants or in the total GDM group vs control participants, stratified by overweight. Further details are shown in ESM Tables 2–9. Differential abundances of non-glucose metabolites were observed only for lipid metabolites in overweight women. Glucose was detected in higher abundance in E-GDM participants (both normal-weight and overweight) (both *q*<0.05).

#### BMI ≥25 kg/m^2^

PC P-16:0/18:2 and PC 15:0/18:2 were detected in lower abundance in E-GDM women compared with BMI-matched control participants (*q*<0.01 and *q*<0.05, respectively). PC 16:0/20:3 was detected in lower abundance in the total GDM group (*q*<0.05). In contrast, lysoPC 20:4/0:0 was increased in the E-GDM subgroup and lysoPC (20:3/0:0) was increased in the total GDM group (both *q*<0.05). Linolenic acid, docosapentaenoic acid (FA C22:5), oleic acid and docosatetraenoic acid (FA C22:4) abundances were increased in the E-GDM group (*q*<0.05, *q*<0.05, *q*<0.05 and *q*<0.01, respectively). Further, oleic acid and docosatetraenoic acid (FA C22:4) showed higher abundances in overweight control participants compared with normal-weight control participants (both *q*<0.05). Linoleic acid was increased in overweight women in the total GDM group vs overweight control participants (*q*<0.05).

### Early-pregnancy metabolic fingerprints of GDM subtypes

**Fig. 5:**
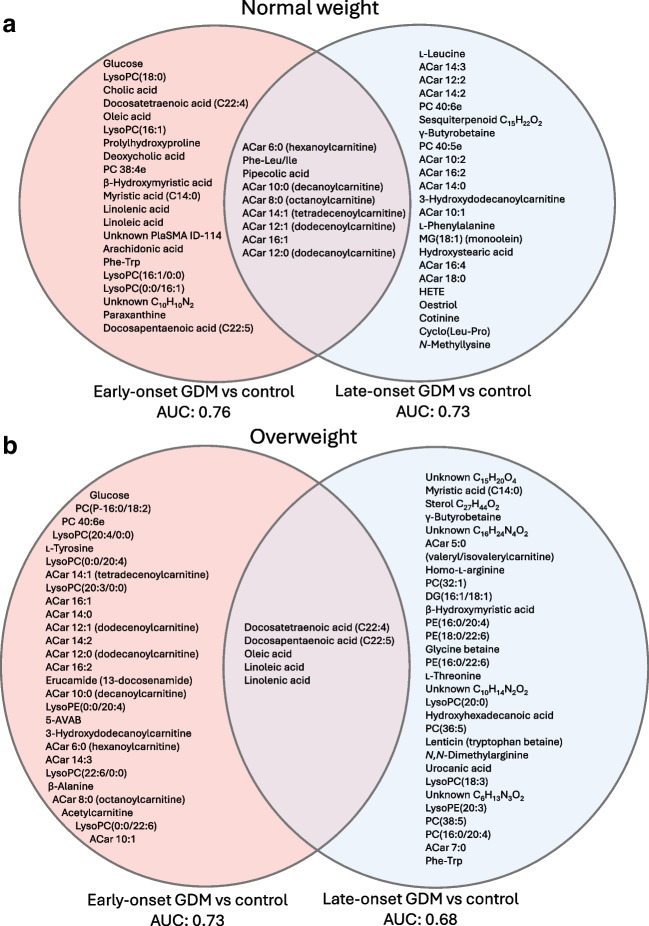
Venn diagrams of metabolites included in PLS-DA models that discriminate E-GDM and L-GDM patients from control participants for both normal-weight (**a**) and overweight (**b**) women. Subgroup-specific AUC estimates are reported for the best-fitting models. 5-AVAB, 5-aminovaleric acid betaine; DG, diacylglycerol; HETE, hydroxyeicosatetraenoic acid; MG, monoglyceride; PE, phosphatidyl ethanolamine

Finally, we applied a multivariate PLS-DA model to explore early-pregnancy metabolite patterns or ‘metabolic fingerprints’ that best differentiate GDM groups (ESM Tables 10–17). Metabolites that predict differences between normal-weight and overweight women with E-GDM or L-GDM vs control participants are shown in Fig. 5. The clearest patterns showed clustering of medium- and long-chain ACar in normal-weight women with E-GDM or L-GDM (Fig. 5a) and overweight women with E-GDM (Fig. 5b), and clustering of long-chain fatty acids and phospholipids in normal-weight women with E-GDM (Fig. 5a) and overweight women with E-GDM or L-GDM (Fig. 5b).

## Discussion

The results of our nested case–control study within the Finnish population-based EDDIE cohort demonstrate that the maternal early-pregnancy metabolome differs in early- and late-onset GDM subtypes and is significantly modified by maternal overweight. At 12–16 weeks’ gestation, the clearest correlation patterns were detected between (1) maternal OGTT glucose concentrations and long-chain fatty acids and ACar, (2) maternal adiposity and long-chain fatty acids, phospholipids, ACar, AAA, BCAA and other amino acids, and (3) indicators of insulin resistance and l-tyrosine, specific long-chain fatty acids and phospholipids. All the differential non-glucose metabolites identified in univariate analyses comparing GDM subtypes were lipid metabolites in women with overweight and GDM, with most alterations detected in long-chain fatty acids and phospholipids in E-GDM. Univariate analyses did not identify statistically significant differences in non-glucose metabolites between normal-weight women with E-GDM or L-GDM vs control participants. However, multivariate analyses comparing the ‘metabolomic fingerprints’ of GDM subgroups vs control participants suggested differences in ACar in normal-weight GDM women and long-chain fatty acids in women with overweight and GDM, regardless of time of GDM onset. E-GDM subtypes in both normal-weight and overweight women showed differences in both ACar and long-chain fatty acids.

To our knowledge, this is the first study to analyse associations between early-pregnancy metabolites and maternal glycaemic and anthropometric traits, and to identify early-pregnancy metabolomic differences between participants with E-GDM and L-GDM, stratified by maternal overweight. Access to the large, population-based EDDIE cohort enabled selection of participants from a mixed ‘real-world’ pool of low- and high-risk pregnant women and the matching of GDM and control participants. All OGTTs were performed in the same laboratory, using standard test protocols [20]. Many previous metabolomic studies have exploited targeted methods, but we used unbiased high-performance LC-MS/MS, enabling wide-scale screening of differential metabolites. One limitation of our study is that the unselected EDDIE cohort included few severely obese participants, which prevented us from studying the various classes of obesity separately. Moreover, although our sample size is more robust than in most previous untargeted LC-MS/MS studies on GDM, a larger sample size would probably enable the detection of more subtle early-pregnancy alterations in GDM subgroups, such as normal-weight women with GDM. The use of an ethnically homogenous study population may also affect the generalisability of our results.

Few previous metabolomic studies have considered the heterogeneity of GDM [17]. A Finnish study using NMR spectroscopy reported metabolomic changes across gestation in obese women with GDM, obese women without GDM and non-obese women with GDM compared with non-obesed women without GDM [28]. However, only women from high-risk obstetric cohorts were included, and metabolomic differences between E-GDM and L-GDM were not explored [28]. Similarly, Lee et al demonstrated differences in the metabolomic and genetic architecture between insulin-sensitive vs insulin-resistant L-GDM subtypes; however, in their study, blood samples were collected at approximately 28 weeks’ gestation [18].

Metabolomic changes involving BCAA and AAA characterise obesity, insulin resistance and type 2 diabetes in non-pregnant individuals [10, 11, 29]. Findings in pregnant women have been inconsistent [30]. Increased levels of BCAA in early pregnancy have been reported in obese women [15] and lean women [31] who develop L-GDM. In contrast, a targeted metabolomics study performed in the first trimester did not observe BCAA differences between women with L-GDM and control participants [32]. Another recent study found that BCAA and AAA concentrations at 15–26 weeks’ gestation, but not at 10–14 weeks’ gestation, were positively associated with L-GDM risk [33]. Similarly, we did not discover significant differences in early-pregnancy BCAA or AAA between E-GDM women or L-GDM women vs control participants in univariate analyses. However, maternal BMI and/or WC correlated positively with AAA (tyrosine and phenylalanine) and BCAA (leucine and valine), and HOMA-IR and fasting insulin correlated with l-tyrosine. Moreover, in PLS-DA models, l-leucine and l-phenylalanine were observed to be differential metabolites in normal-weight women with L-GDM, and l-tyrosine was a differential metabolite in overweight women with E-GDM. Previous studies have also linked these amino acids to insulin resistance in early pregnancy [34] and late pregnancy [18, 35], and to L-GDM in obese women [15].

Exaggerated late-pregnancy dyslipidaemia and alterations in maternal, placental and fetal fatty acid metabolism have been reported in GDM and in pregnancies in women with obesity [15, 36]. Data on lipid metabolism in early pregnancy are less abundant. We observed increased early-pregnancy levels of several long-chain fatty acids, e.g. oleic acid, docosapentaenoic acid, docosatetraenoic acid, and linolenic acid, particularly in women with E-GDM. These fatty acids correlated most strongly with post-load glucose concentrations, maternal BMI and WC, and emerged as differential metabolites characterising the early-pregnancy metabolomic profiles of women with E-GDM and overweight GDM vs control participants. These results agree with studies showing elevated levels of long-chain fatty acids, such as oleic acid and linoleic acid, in non-pregnant obese adults [37] and in early pregnancy in women who develop L-GDM [38]. Elevated docosapentaenoic acid levels, on the other hand, have been previously associated with reduced L-GDM risk when measured in early pregnancy [39], although an increased risk of the metabolic syndrome has been reported in non-pregnant individuals [40].

Both obesity and diabetes are associated with derangements of sphingolipid, phospholipid and lysolipid pathways [41]. The association of sphingomyelin d32:2 with maternal overweight in early pregnancy and its correlation with maternal BMI in our cohort confirm earlier findings [34]. Our observations of reduced early-pregnancy abundance of various PC species in overweight and/or GDM women, and the negative correlations of several PC species with BMI and WC, are also in concordance with the results of previous studies [14, 42]. Regarding lysoPCs, studies in non-pregnant individuals have shown reduced levels in relation to obesity [37, 43] and type 2 diabetes [11, 43]. Studies in lean pregnant women [44, 45] and obese pregnant women [42] have yielded less consistent results, suggesting both positive and negative associations between levels of lysoPC species in early pregnancy and L-GDM. In agreement with this, we observed negative correlations between lysoPC 20:0 and maternal BMI and WC, but positive correlations between lysoPC 16:1 and parameters of adiposity and insulin resistance. Moreover, higher abundances of lysoPC 20:4/0:0 were identified in overweight women with E-GDM, whereas lysoPC 20:3/0:0 showed higher abundance among overweight women in the total GDM group in univariate analyses and correlated with post-load glucose, maternal WC and HOMA-IR. Hence, our findings support the involvement of lysoPCs in the metabolomic alterations of early pregnancy that characterise E-GDM and perhaps also L-GDM, but the gestational timepoint and maternal BMI may influence species-specific directions of associations.

Short- and long-chain ACar are associated with type 2 diabetes risk [10, 11]. While short-chain ACar are linked to BCAA catabolism, long-chain ACar may result from incomplete fatty acid oxidation, contributing to insulin resistance [46]. A longitudinal cohort study reported associations between increased levels of medium-chain ACar (C14:1-OH) at 10–14 weeks’ gestation and L-GDM [46]. Likewise, in our whole cohort, medium- and long-chain ACar were elevated in GDM and overweight women, and correlated positively with post-load glucose, maternal BMI and WC. Although we did not observe differences in ACar between GDM subgroups in univariate analyses, ACar emerged as differential metabolites in normal-weight women with E-GDM and L-GDM, and in overweight women with E-GDM, in multivariate PLS-DA models. This finding is interesting given that Lee et al reported that several ACar in late pregnancy were associated with insulin-deficient GDM [18].

We observed the most extensive metabolomic perturbations in overweight women with E-GDM, in accordance with reports showing high risks of perinatal complications in insulin-resistant [4], early-onset [7] and overweight [6, 47] GDM subtypes. In contrast, our data suggest fewer early-pregnancy metabolomic differences between overweight women with L-GDM vs overweight control participants, as supported by the lowest AUC value in the PLS-DA model. For normal-weight women with GDM, we were surprised that only glucose emerged as an altered metabolite in E-GDM women vs control participants, and that the metabolome of L-GDM women did not statistically significantly differ from that of BMI-matched control participants at 12–16 weeks’ gestation in univariate analyses. It is plausible that, in lean women with GDM, genetic risk factors and beta cell dysfunction play a greater pathogenetic role than chronic insulin resistance. Although most known GDM risk alleles identified are variants that associate with beta cell function, a recent genome-wide association study reported genetic risk alleles for GDM in central glucose homeostasis, steroidogenesis and placental expression [48]. It is noteworthy that, in our study, normal-weight women with E-GDM showed elevated fasting insulin concentrations and HOMA-IR compared with normal-weight and overweight normoglycaemic control participants, suggesting some degree of early-pregnancy insulin resistance. This is in line with previous observations indicating deficient first-trimester beta cell function and substantial reduction in early-pregnancy insulin sensitivity in women with E-GDM [49].

In conclusion, our untargeted LC-MS metabolomic analyses revealed specific early-pregnancy metabolome differences between lean and overweight women with E-GDM and L-GDM. Most metabolomic alterations were detected in overweight women with E-GDM and involved lipid metabolites that correlated with post-glucose concentrations, maternal BMI and WC, underscoring the importance of maternal pre-conception weight management.

## Supplementary Information

Below is the link to the electronic supplementary material.

ESM (PDF 812 KB)

## Data Availability

The data that support the findings of this study are not openly available for reasons of sensitivity, but may be available from the corresponding author upon reasonable request.

## Abbreviations

ACar: Acylcarnitine(s)
AAA: Aromatic amino acids
BCAA: Branched-chain amino acids
EDDIE: Early diagnosis of diabetes in pregnancy
E-GDM: Early gestational diabetes mellitus
FA: Fatty acid
GDM: Gestational diabetes mellitus
L-GDM: Late gestational diabetes mellitus
LysoPC: Lysophosphatidylcholine
PC: Phosphatidylcholine
Phe-Trp: Phenylalanyltryptophan
WC: Waist circumference
