# Supplementary material for: Analysis of early-pregnancy metabolome in early- and late-onset gestational diabetes reveals distinct associations with maternal overweight
Source: Diabetologia. 2024 Jul 31;67(11):2539–54. doi: 10.1007/s00125-024-06237-x (PMC11519293; doi:10.1007/s00125-024-06237-x)
Supplement: Supplementary file 1 — ESM (PDF 812 KB) [file 125_2024_6237_MOESM1_ESM.pdf]

Electronic supplementary materials (ESM)

ESM Methods

Analysis of HbA_{1c}, plasma glucose, and serum insulin concentrations

HbA_{1c} was analyzed from fresh venous blood samples, drawn into K2-EDTA blood collection tubes, using a quantitative latex agglutination inhibition method (Siemens Advia 1800 analyzer), in line with the recommendations of the International Federation of Clinical Chemistry. Plasma glucose during oral glucose tolerance tests (OGTTs) was analyzed from 2mL fresh venous blood samples, drawn into citrate-fluoride blood collection tubes. Shortly after the blood draw, whole blood was separated into packed red cells, buffy coat, and plasma by centrifuging at 2540 RCF for 15 min at room temperature. Plasma glucose was determined at the SKCH laboratory with a photometric hexokinase method (Siemens Advia 1800 analyzer) within 5 hours from the time of venipuncture. Fasting serum insulin was analyzed at Vita Laboratories, Helsinki, from venous blood samples collected in conjunction with OGTT1 at 12-16 weeks' gestation, initially frozen at -80C, and thawed once for the analyses. An electrochemiluminescence immunoassay (ECLIA) method was used for the analysis of serum insulin. Fasting serum insulin was analyzed from venous blood samples drawn in conjunction with OGTT1. For these analyses, whole blood was centrifuged at 2540 RCF for 15 min at 4°C, and serum was initially frozen at -80C in aliquots of 1mL. The samples were thawed once for the analysis of insulin concentrations by the electrochemiluminescence immunoassay (ECLIA) method. Fasting serum insulin was in mU/l and converted to pmol/l using a conversion factor of 6.945, using the AMA Manual of Style 11th Edition: a guide for authors and editors conversion calculator (available from https://academic.oup.com/amamanualofstyle/si-conversiov–calculator; accessed 14 May 2024). Insulin resistance was quantified using the Homeostasis Model Assessment of Insulin Resistance (HOMA-IR) (Matthews DR et al 1985, https://doi.org/10.1007/BF00280883).

Sample preparation for LC-MS analysis

100 µL aliquots of the serum samples were mixed with 400 µL of acetonitrile by pipetting. The samples were centrifuged at 18 000 × g for 10 min at 4 °C and filtered through 0.2-µm polytetrafluoroethylene filters into a 96-well plate. Small aliquots (2–5 µL) were taken from each sample, mixed in a single tube, and used as the quality control (QC) sample in the analysis.

Data analysis

After the peak picking, a total of 33 352 molecular features was included in the data preprocessing and clean-up step. Low-quality features were flagged and discarded from statistical analyses. Molecular features were only kept if they met all the following quality metrics: low number of missing values, present in more than 70% of the QC samples, RSD* below 20%, D-ratio* below 40%. In addition, if either RSD* or D-ratio* was above the threshold, the features were still kept if their classic RSD, RSD*, and basic D-ratio were all below 10%. The signals were normalized for signal drift using feature-wise cubic spline regression on log-transformed abundance values and for batch effect using correction presented in the batchCorr R package (Brunius et al 2016, https://doi.org/10.1007/s11306-016-1124-4).
Missing values were imputed using random forest imputation for high-quality features and zero imputation for low-quality features.

The high number of molecular features before data clean-up is due to the high sensitivity of the instrument, collecting several signals from each actual metabolite, but also from the solvent background and detector noise.

Notes about the multivariate model

Feature-wise statistical analyses can miss interactions between molecular features and can highlight multiple intercorrelated features. Multivariate analyses provide a different point of

view on the data structure and complement feature-wise analyses (Worley and Powers, 2013: https://doi.org/10.2174/2213235X11301010092). A challenge in using multivariate methods with feature selection on untargeted metabolomics data is that the data often contains multiple strongly correlated features (possibly originating from the same compound), of which only some can be identified. If the feature selection process favors a feature that cannot be identified over a strongly correlated feature that can be identified, some important information may be lost. To circumvent this problem, we used a feature clustering approach, presented by Klåvus et al (2020) (https://doi.org/10.3390/metabo10040135), to cluster similar features together before multivariate modeling. Briefly, the process groups together features that originate from the same analytical mode, are strongly correlated, and have a small difference in their retention time. The feature with the largest median peak area represents the cluster in the multivariate model. We then assigned the feature importance score of the cluster to each feature in the cluster to guide feature identification.

For multivariate analysis, we used partial least-squares discriminant analysis (PLS-DA), with unbiased cross-validation and feature selection procedures from the MUVR R package (version 0.0.974; Department of Biology and Biological Engineering, Chalmers University of Technology, Sweden; Shi et al 2019, https://doi.org/10.1093/bioinformatics/bty710). We fit binary classification models analogous to the feature-wise statistics for the difference between two gestational diabetes (GDM) subgroups. As in feature-wise statistics, the data was split into normal-weight (BMI <25 kg/m^2) and overweight (BMI ≥25 kg/m^2) participants before each analysis. The compared pairs of groups were as follows: total GDM group *vs*. controls, early-onset GDM *vs*. controls, late-onset GDM *vs*. controls, and early-onset GDM vs. late-onset GDM. This resulted in a total of 8 models fit. We used the “mid” option from MUVR to find the best-performing model for each comparison and recorded the features selected for each

model. This information was used to prioritize feature identification along with the feature-wise statistics.

After feature identification, we fit the final PLS-DA models with MUVR using only molecular features we could annotate, to see how well the annotated features represent the molecular fingerprint of the differences between the GDM subgroups. We report the feature importance from these models along with the area under receiver operating characteristic curve (AUC) values to showcase the predictive performance of each model. All the models except "total GDM *vs*. control in normal-weight" had an AUC >0.7 (see Table below), which we considered acceptable (Mandrekar 2010, https://doi.org/10.1097/jto.0b013e3181ec173d) and a sign that the models represent part of the molecular fingerprint of GDM. We acknowledge that the AUC results might be positively biased since we performed feature selection using the complete dataset and evaluated performance using cross-validation instead of an external dataset. However, the double-cross-validation process in the MUVR process aims to limit this bias (Shi et al 2019, https://doi.org/10.1093/bioinformatics/bty710). The features chosen with feature-wise statistics still induce this bias, but including only annotated features in the model compensates for this issue.

**Model**	**AUC**
Total GDM *vs*. control in normal weight	0.589855
Total GDM *vs*. control in overweight	0.709125
Early-onset GDM *vs*. control in normal weight	0.738192
Late-onset GDM *vs*. control in normal weight	0.727952
Late-onset GDM *vs*. early-onset GDM in normal weight	0.75052
Early-onset GDM *vs*. control in overweight	0.771073
Late-onset GDM *vs*. control in overweight	0.708736
Late-onset GDM vs. early-onset GDM in overweight	0.773294

ESM Table 1. Differential metabolites (*n* = 162) in at least one of the 15 univariate tests ($p<0.05$). The table includes information about the biochemical classification (ontology), Human Metabolome Database (HMDB) entry ID, chromatographic and ion mode, type of adduct ion, retention time (RT) in minutes, mass-to-charge ratio (*m/z*), and reliability of identification (ID level) of the metabolites. ID level according to Sumner et al. (2007) (https://doi.org/10.1007/s11306-007-0082-2): 1 = identified based on a reference standard, 2 = putatively annotated based on MS/MS spectra or physicochemical properties, 3 = putatively characterized compound class based on spectral similarity.

Metabolite	Ontology	HMDB ID	Mode	Adduct	RT [min]	*m/z*	ID level
3-Hydroxydodecanoylcarnitine	Acylcarnitine		HILIC+	[M+H]+	0.97	360.28	2
ACar 10:0 (decanoylcarnitine)	Acylcarnitine	HMDB0000651	RP+	[M+H]+	7.33	316.25	1
ACar 10:1	Acylcarnitine		RP+	[M]+	6.86	314.23	2
ACar 10:2	Acylcarnitine		RP+	[M]+	6.35	312.22	2
ACar 11:1	Acylcarnitine		RP+	[M]+	7.31	328.25	2
ACar 12:0 (dodecanoylcarnitine)	Acylcarnitine	HMDB0002250	RP+	[M+H]+	8.21	344.28	1
ACar 12:1 (dodecenoylcarnitine)	Acylcarnitine		RP+	[M]+	7.81	342.26	1
ACar 12:2	Acylcarnitine		RP+	[M]+	7.49	340.25	2
ACar 14:0	Acylcarnitine	HMDB0254979	RP+	[M]+	8.87	372.31	1
ACar 14:1 (tetradecenoylcarnitine)	Acylcarnitine		RP+	[M]+	8.55	370.30	1
ACar 14:2	Acylcarnitine		RP+	[M]+	8.17	368.28	2
ACar 14:3	Acylcarnitine		RP+	[M]+	7.78	366.26	2
ACar 16:1	Acylcarnitine		RP+	[M]+	9.05	398.33	2
ACar 16:2	Acylcarnitine		RP+	[M]+	8.74	396.31	2
ACar 16:4	Acylcarnitine		RP+	[M]+	8.21	392.28	2
ACar 18:1	Acylcarnitine		RP+	[M+H]+	9.49	426.36	2
ACar 18:3	Acylcarnitine		RP+	[M]+	8.96	422.33	2
ACar 5:0 (valeryl/isovalerylcarnitine)	Acylcarnitine		HILIC+	[M+H]+	1.24	246.17	2
ACar 6:0 (hexanoylcarnitine)	Acylcarnitine	HMDB0000756	RP+	[M+H]+	4.26	260.19	2
ACar 7:0	Acylcarnitine	HMDB0013238	HILIC+	[M]+	0.91	274.20	2
ACar 8:0	Acylcarnitine	HMDB0000791	HILIC+	[M+Na]+	0.83	310.20	1
ACar 8:0 (octanoylcarnitine)	Acylcarnitine	HMDB0000791	RP+	[M+H]+	6.07	288.22	1
ACar 8:1	Acylcarnitine		HILIC+	[M+H]+	0.92	286.20	2
Acetylcarnitine	Acylcarnitine	HMDB0000201	HILIC+	[M]+	2.80	204.12	1
ACar 8:1 (octenoylcarnitine)	Acylcarnitine		RP+	[M+H]+	5.26	286.20	2
Cotinine	Alkaloid	HMDB0001046	HILIC+	[M+H]+	0.54	177.10	2
Paraxanthine	Alkaloid	HMDB0001860	RP+	[M+H]+	2.77	181.07	1

Metabolite	Ontology	HMDB ID	Mode	Adduct	RT [min]	*m/z*	ID level
β-Alanine	Amino acid	HMDB0000056	HILIC+	[M+H]+	5.64	90.06	2
Cystine	Amino acid	HMDB0000192	HILIC+	[M+H]+	7.67	241.03	1
Homo-L-arginine	Amino acid	HMDB0000670	HILIC+	[M+H]+	6.83	189.13	2
L-Glutamine	Amino acid	HMDB0000641	HILIC+	[M+H]+	6.12	147.08	1
L-Histidine	Amino acid	HMDB0000177	HILIC+	[M+H]+	6.79	156.08	1
L-Leucine	Amino acid	HMDB0000687	HILIC+	[M+H]+	4.05	132.10	1
L-Phenylalanine	Amino acid	HMDB0000159	HILIC+	[M+H]+	4.03	166.09	1
L-Proline	Amino acid	HMDB0000162	HILIC+	[M+H]+	4.81	116.07	1
L-Threonine	Amino acid	HMDB0000167	HILIC+	[M+H]+	5.78	120.07	1
L-Tryptophan	Amino acid	HMDB0000929	HILIC+	[M+H]+	4.03	205.10	1
L-Tyrosine	Amino acid	HMDB0000158	HILIC+	[M+H]+	5.10	182.08	1
L-Valine	Amino acid	HMDB0000883	HILIC+	[M+H]+	4.83	118.09	2
N,*N*-Dimethylarginine	Amino acid	HMDB0251395	RP+	[M+H]+	0.58	203.15	2
N-Methyllysine	Amino acid	HMDB0002038	HILIC+	[M+H]+	6.80	161.13	2
Androgen C19H30O	Androgen		RP+	[M+H-H2O]+	10.09	257.23	3
5-Aminovaleric acid betaine (5-AVAB)	Betaine	HMDB0240732	HILIC+	[M+H]+	1.97	160.13	1
gamma-Butyrobetaine	Betaine	HMDB0001161	HILIC+	[M+H]+	3.07	146.12	1
Glycine betaine	Betaine	HMDB0000043	HILIC+	[M+H]+	3.58	118.09	1
Lenticin (tryptophan betaine)	Betaine	HMDB0061115	HILIC+	[M+H]+	1.39	247.14	2
Bilirubin	Bile pigment	HMDB0000054	RP+	[M+H]+	5.96	585.27	2
Bilirubin (isomer 2)	Bile pigment	HMDB0000054	RP-	[M-H]-	7.18	583.26	2
PE-Cer(d34:1)	Ceramide phosphoethanolamine		RP+	[M+H]+	12.02	661.53	3
Cyclo(Leu-Pro)	Cyclic peptide	HMDB0034276	RP+	[M+H]+	4.72	211.14	2
DG(16:1/18:1)	Diacylglycerol	HMDB0007130	RP+	[M+NH4]+	14.77	610.54	2
Octadecanedioic acid	Dicarboxylic acid	HMDB0000782	RP-	[M-H]-	10.15	313.24	2
Phe-Leu/Ile	Dipeptide		RP+	[M+H]+	4.59	279.17	2
Phe-Trp	Dipeptide	HMDB0029006	RP+	[M+Na]+	4.75	352.16	2
Prolylhydroxyproline	Dipeptide	HMDB0006695	HILIC+	[M+H]+	6.11	229.12	2
HETE	Eicosanoid		RP-	[M-H]1-	9.96	319.23	3
Estriol	Estrogen steroid	HMDB0000153	RP+	[M+H]+	7.57	271.21	2
Tetradecanoic acid (C14:0)	Fatty acid	HMDB0000806	RP+	[M+H]+	11.14	228.20	2
FAHFA 34:2	Fatty acid ester of hydroxy fatty acid		RP-	[M-H]-	11.16	533.46	3
FAHFA 18:0	Fatty acid ester of hydroxyl fatty acid		RP-	[M-H]-	11.13	313.24	2
Erucamide (13-docosenamide)	Fatty amide	HMDB0244507	RP+	[M+H]+	11.08	338.34	2
Urocanic acid	Histidine metabolite	HMDB0000301	HILIC+	[M+H]+	1.04	139.05	1
3-Hydroxybutanoic acid	Hydroxy acid	HMDB0000011	HILIC-	[M-H]-	0.70	103.04	2
β-Hydroxymyristic acid	Hydroxy fatty acid	HMDB0061656	RP-	[M-H]-	9.90	243.20	2
Hydroxystearic acid	Hydroxy fatty acid	HMDB0062549	RP-	[M-H]-	11.10	299.26	2

Metabolite	Ontology	HMDB ID	Mode	Adduct	RT [min]	*m/z*	ID level
3-Indolepropionic acid	Indole	HMDB0002302	RP+	[M+H]+	6.08	190.09	1
Indoleacetaldehyde	Indole	HMDB0001190	RP+	[M+H-2H2O]+	2.92	160.08	2
Unknown lipid C20H30O2	Lipid		RP+	[M+H]+	10.00	303.23	4
Pipecolic acid	Lysine metabolite	HMDB0000070	HILIC+	[M+H]+	4.95	130.09	1
LysoPA(20:4)	Lysophosphatidic acid	HMDB0114742	RP-	[M-H]-	11.88	457.24	2
LysoPA(22:4)	Lysophosphatidic acid	HMDB0114752	RP-	[M-H]-	11.97	457.24	2
LysoPC(16:1)	Lysophosphatidylcholine	HMDB0010383	HILIC+	[M+H]+	1.26	494.32	2
LysoPC(0:0/16:1)	Lysophosphatidylcholine	HMDB0010383	RP+	[M+H]+	9.99	494.32	2
LysoPC(0:0/20:4)	Lysophosphatidylcholine	HMDB0061699	RP-	[M+FA-H]-	10.19	588.33	2
LysoPC(0:0/22:6)	Lysophosphatidylcholine	HMDB0010404	RP+	[M+H]+	10.20	568.34	2
LysoPC(16:1/0:0)	Lysophosphatidylcholine	HMDB0010383	RP+	[M+H]+	10.13	494.32	2
LysoPC(18:0)	Lysophosphatidylcholine	HMDB0011128	RP+	[M+Na]+	10.51	546.35	2
LysoPC(18:3)	Lysophosphatidylcholine		HILIC+	[M+Na]+	1.26	518.32	2
LysoPC(20:0)	Lysophosphatidylcholine	HMDB0010390	RP+	[M+H]+	11.35	552.40	2
LysoPC(20:1)	Lysophosphatidylcholine	HMDB0010391	RP+	[M+H]+	11.02	550.39	2
LysoPC(20:3/0:0)	Lysophosphatidylcholine	HMDB0010393	RP-	[M+FA-H]-	10.51	590.35	2
LysoPC(20:4/0:0)	Lysophosphatidylcholine	HMDB0010395	RP+	[M+H]+	10.31	544.34	2
LysoPC(22:4)	Lysophosphatidylcholine	HMDB0010401	RP-	[M+FA-H]-	10.68	616.36	2
LysoPC(22:5)	Lysophosphatidylcholine	HMDB0010402	RP+	[M+H]+	10.45	570.36	2
LysoPC(22:6/0:0)	Lysophosphatidylcholine	HMDB0010404	RP+	[M+H]+	10.30	568.34	2
LysoPE(0:0/20:4)	Lysophosphatidylethanolamine	HMDB0011487	RP-	[M-H]-	10.20	500.28	2
LysoPE(18:2)	Lysophosphatidylethanolamine		RP+	[M+H]+	10.22	478.29	2
LysoPE(20:3)	Lysophosphatidylethanolamine		RP-	[M-H]-	10.50	502.29	2
LysoPE(20:4)	Lysophosphatidylethanolamine	HMDB0011517	RP+	[M+H-H2O]+	10.22	502.29	2
LysoPE(22:6)	Lysophosphatidylethanolamine		RP+	[M+H]+	10.21	526.29	2
MG(18:1) (monoolein)	Monoacylglycerol	HMDB0011567	RP+	[M+H]+	10.96	357.30	2
MG(18:0)	Monoacylglycerol	HMDB0011131	RP+	[M+H]+	11.28	359.32	2
Oleic acid	MUFA	HMDB0000207	RP-	[M-H]-	11.26	281.25	2
1-Methylnicotinamide	Nicotinamide	HMDB0000699	HILIC+	[M]+	2.11	137.07	1
Paracetamol	Pharmaceutical	HMDB0001859	RP+	[M+H]+	2.50	152.07	2
PC 40:8	Phosphatidylcholine		RP+	[M+H]+	12.72	830.57	3
PC(14:0/16:0)	Phosphatidylcholine	HMDB0007869	RP-	[M-H]-	12.91	750.53	2
PC(15:0/18:2)	Phosphatidylcholine		RP+	[M+K]+	12.92	744.55	2
PC(16:0/18:2)	Phosphatidylcholine		RP+	[M+H]+	13.25	758.57	2
PC(16:0/20:4)	Phosphatidylcholine	HMDB0007982	RP-	[M-H]-	13.13	826.56	2
PC(17:0/18:2)	Phosphatidylcholine		RP+	[M+H]+	13.67	772.58	2
PC(18:0/22:6)	Phosphatidylcholine	HMDB0008057	RP-	[M+Cl]-	13.80	878.59	2

**Metabolite**	**Ontology**	**HMDB ID**	**Mode**	**Adduct**	**RT [min]**	***m/z***	**ID level**
PC(18:2/18:2)	Phosphatidylcholine	HMDB0008138	RP-	[M+FA-H]-	12.89	826.56	2
PC(32:1)	Phosphatidylcholine		RP+	[M+H]+	13.06	732.56	3
PC(32:2)	Phosphatidylcholine		RP+	[M+K]+	12.62	730.54	3
PC(33:1)	Phosphatidylcholine		RP+	[M+Na]+	13.42	746.57	3
PC(16:0/20:3)	Phosphatidylcholine		RP+	[M+H]+	13.40	784.59	2
PC(18:0/20:4)	Phosphatidylcholine		RP+	[M+H]+	13.99	810.60	2
PC(36:5)	Phosphatidylcholine		RP+	[M+H]+	12.54	780.55	3
PC(38:5)	Phosphatidylcholine		RP-	[M+FA-H]-	13.28	852.58	2
PE(16:0/18:2)	Phosphatidylethanolamine	HMDB0008928	RP-	[M-H]-	13.18	714.51	2
PE(16:0/20:4)	Phosphatidylethanolamine	HMDB0008937	RP-	[M-H]-	13.09	738.51	2
PE(16:0/22:6)	Phosphatidylethanolamine		RP-	[M-H]-	12.98	762.51	2
PE(18:1/20:4)	Phosphatidylethanolamine	HMDB0009036	RP-	[M-H]-	13.22	764.52	2
PE(36:3)	Phosphatidylethanolamine		RP+	[M+H]+	13.35	742.54	3
PS(O-20:0/0:0)	Phosphatidylserine		RP+	[M+K]+	9.53	540.37	2
2-Piperidinone	Piperidine	HMDB0011749	HILIC+	[M+H]+	0.57	100.08	2
PC(P-16:0/18:2)	Plasmalogen		RP+	[M+H]+	13.62	742.57	2
PC(O-16:0/18:2)	Plasmalogen		RP+	[M+H]+	13.82	744.59	3
PC 36:3e	Plasmalogen		RP+	[M+H]+	13.98	770.61	3
PC 38:4e	Plasmalogen		RP+	[M+H]+	14.75	796.62	3
PC 38:6e	Plasmalogen		RP+	[M+H]+	13.35	812.56	3
PC 40:5e	Plasmalogen		RP+	[M+H]+	15.17	822.63	2
PC 40:6e	Plasmalogen		RP+	[M+H]+	13.99	820.62	3
PC(P-18:0/22:6)	Plasmalogen		RP+	[M+H]+	13.69	818.61	3
PE(P-16:0/22:6)	Plasmalogen	HMDB0005780	RP-	[M-H]-	13.28	746.51	2
Sinapyl alcohol	Polyphenol	HMDB0013070	RP+	[M+H]+	6.51	211.10	1
7alpha-Hydroxy-3-oxo-4-cholestenoic acid	Primary bile acid	HMDB0012458	RP-	[M-H]-	9.97	429.30	2
Cholic acid	Primary bile acid	HMDB0000619	RP+	[M-2H2O+H]+	9.32	373.27	1
Arachidonic acid	PUFA	HMDB0001043	RP-	[M-H]-	10.94	303.23	2
Docosatetraenoic acid (C22:4)	PUFA	HMDB0002226	RP-	[M-H]-	11.28	331.26	2
Docosahexaenoic acid	PUFA	HMDB0002183	RP-	[M-H]-	10.91	327.23	2
Docosapentaenoic acid (C22:5)	PUFA	HMDB0001976	RP-	[M-H]-	11.05	329.25	2
Eicosapentaenoic acid (C20:5)	PUFA	HMDB0001999	RP-	[M-H]-	10.70	301.22	2
Linoleic acid	PUFA	HMDB0000673	RP-	[M-H]-	10.98	279.23	2

Metabolite	Ontology	HMDB ID	Mode	Adduct	RT [min]	*m/z*	ID level
Linolenic acid	PUFA		RP-	[M-H]-	10.73	277.22	3
Myristic acid (C14:0)	SAFA	HMDB0000806	RP-	[M-H]-	10.70	227.20	2
Palmitic acid (C16:0)	SAFA	HMDB0000220	RP-	[M-H]-	11.16	255.23	2
Deoxycholic acid	Secondary bile acid	HMDB0000626	RP+	[M-2H2O+H]+	9.94	357.28	2
Anhydroretinol	Sesquiterpenoid	HMDB0062447	RP+	[M+H-H2O]+	10.87	269.23	2
Sesquiterpenoid C15H22O2	Sesquiterpenoid		RP+	[M+H]+	9.12	235.17	3
SM d32:2	Sphingomyelin		RP+	[M+H]+	11.90	673.53	2
SM d33:1	Sphingomyelin		RP-	[M-H]-	12.52	733.55	2
SM d34:2	Sphingomyelin		RP-	[M+FA-H]-	12.37	745.55	2
SM d42:3	Sphingomyelin		RP-	[M-H]-	14.94	855.66	2
SM(d18:1/12:0)	Sphingomyelin		RP+	[M+H]+	11.80	647.51	2
Dehydroepiandrosterone sulfate (isomer 1)	Steroid sulfate	HMDB0001032	RP-	[M-H]-	7.63	367.16	2
Dehydroepiandrosterone sulfate (isomer 2)	Steroid sulfate	HMDB0001032	RP-	[M-H]-	8.50	367.16	2
Sterol C27H44O2	Sterol		RP+	[M+H]+	11.45	401.34	3
Glucose	Sugar	HMDB0304632	HILIC-	[M-H]-	5.15	179.06	2
Uric acid	Xanthine	HMDB0000289	HILIC-	[M-H]-	4.88	167.02	2
Unknown C10H10N2			HILIC+	[M+H]+	4.03	159.09	4
Unknown C10H14N2O2			RP+	[M+H]+	2.96	195.11	4
Unknown C10H18O3			RP+	[M+CH3OH+H]+	10.27	187.13	4
Unknown C12H16O3			RP+	[M+H]+	8.87	209.12	4
Unknown C15H20O4			RP+	[M+H]+	7.27	265.14	4
Unknown C15H21NO4			HILIC+	[M+H]+	1.25	280.15	4
Unknown C16H24N4O2			HILIC+	[M+H]+	1.39	305.20	4
Unknown C22H28O2			HILIC+	[M+H]+	0.47	325.22	4
Unknown C5H8N2O2			HILIC+	[M+Na]+	6.13	151.05	4
Unknown C6H13N3O2			HILIC+	[M+H]+	2.55	160.11	4
Unknown PlaSMA ID-114			HILIC-	[M-H]-	0.47	153.02	4

ESM Table 2. Differential metabolites ($p<0.05$; $q<0.1$ in bold, $q<0.05$ underlined) between **total GDM** and **control groups** in **normal-weight** individuals. Cohen's *d* signifies the effect size: a positive value means higher average abundance in the case group versus control. The raw *p*-values and Benjamini-Hochberg false discovery rate (FDR) corrected *q* values are shown. Human Metabolome Database (HMDB) entry ID, chromatographic and ion mode, type of adduct ion, retention time (RT) in minutes, mass-to-charge ratio (*m/z*), and reliability of identification (ID level) of the metabolites. ID level according to Sumner et al. 2007 (https://doi.org/10.1007/s11306-007-0082-2): 1 = identified based on a reference standard, 2 = putatively annotated based on MS/MS spectra or physicochemical properties, 3 = putatively characterized compound class based on spectral similarity.

Metabolite	Ontology	HMDB ID	Cohen's *d*	*p* value	*q* value
ACar 6:0 (hexanoylcarnitine)	Acylcarnitine	HMDB0000756	0.5	0.0024657	0.5291991
Phe-Leu/Ile	Dipeptide		0.499	0.0024744	0.5291991
Glucose	Sugar	HMDB0304632	0.487	0.0031345	0.5291991
Docosatetraenoic acid (C22:4)	PUFA	HMDB0002226	0.482	0.0034622	0.5355953
Arachidonic acid	PUFA	HMDB0001043	0.451	0.0061594	0.5717884
LysoPC(18:0)	Lysophosphatidylcholine	HMDB0011128	0.448	0.0064906	0.5717884
Phe-Trp	Dipeptide	HMDB0029006	0.441	0.0073133	0.5717884
Pipecolic acid	Lysine metabolite	HMDB0000070	0.436	0.0080594	0.5717884
MG(18:1) (monoolein)	Monoacylglycerol	HMDB0011567	0.421	0.0104423	0.5732581
LysoPC(16:1)	Lysophosphatidylcholine	HMDB0010383	0.414	0.0117737	0.5801371
ACar 8:0 (octanoylcarnitine)	Acylcarnitine	HMDB0000791	0.397	0.0154450	0.6159364
Unknown C16H24N4O2			-0.397	0.0156277	0.6159364
ACar 10:1	Acylcarnitine		0.396	0.0156977	0.6159364
ACar 10:0 (decanoylcarnitine)	Acylcarnitine	HMDB0000651	0.391	0.0172536	0.6159364
ACar 12:1 (dodecenoylcarnitine)	Acylcarnitine		0.388	0.0179358	0.6159364
N-Methyllysine	Amino acid	HMDB0002038	-0.37	0.0240908	0.6373105
L -Glutamine	Amino acid	HMDB0000641	-0.369	0.0244267	0.6373105
LysoPC(0:0/16:1)	Lysophosphatidylcholine	HMDB0010383	0.367	0.0251114	0.6414050
LysoPC(16:1/0:0)	Lysophosphatidylcholine	HMDB0010383	0.365	0.0259178	0.6414050
Linolenic acid	PUFA		0.364	0.0264146	0.6414050
PC 38:4e	Plasmalogen		-0.354	0.0305522	
Oleic acid	MUFA	HMDB0000207	0.353	0.0311044	0.6756875
ACar 14:1 (tetradecenoylcarnitine)	Acylcarnitine		0.352	0.0316499	0.6756875
Cholic acid	Primary bile acid	HMDB0000619	0.346	0.0344206	0.6757085
Prolylhydroxyproline	Dipeptide	HMDB0006695	-0.343	0.0358727	0.6757085
Cystine	Amino acid	HMDB0000192	-0.343	0.0362801	0.6757085
PC 40:6e	Plasmalogen		-0.34	0.0375366	
LysoPC(22:4)	Lysophosphatidylcholine	HMDB0010401	0.338	0.0387150	0.6761946

Metabolite	Ontology	HMDB ID	Cohen's *d*	*p* value	*q* value
Paraxanthine	Alkaloid	HMDB0001860	0.337	0.0393992	0.6761946
ACar 16:1	Acylcarnitine		0.336	0.0401068	0.6761946
PS(O-20:0/0:0)	Phosphatidylserine		-0.327	0.0453982	0.6908334
Sesquiterpenoid C15H22O2	Sesquiterpenoid		-0.327	0.0454847	0.6908334
PC 38:6e	Plasmalogen		-0.327	0.0456857	
LysoPA(20:4)	Lysophosphatidic acid	HMDB0114742	0.327	0.0458186	0.6908334
PC(P-16:0/18:2)	Plasmalogen		-0.323	0.0483805	0.6988312
ACar 12:0 (dodecanoylcarnitine)	Acylcarnitine	HMDB0002250	0.323	0.0485057	0.6988312
Unknown C5H8N2O2			-0.322	0.0490471	0.7001312
ACar 14:3	Acylcarnitine		0.322	0.0492940	0.7021495

ESM Table 3. Differential metabolites ($p< 0.05$, $q<0.1$ in bold, $q<0.05$ underlined) between **total GDM** and **control groups** in **overweight** individuals.

Metabolite	Ontology	HMDB ID	Cohen's *d*	*p* value	*q* value
Docosatetraenoic acid (C22:4)	PUFA	HMDB0002226	0.587	0.0000028	**<u>0.0007446</u>**
Oleic acid	MUFA	HMDB0000207	0.504	0.0000510	**<u>0.0046512</u>**
Docosapentaenoic acid (C22:5)	PUFA	HMDB0001976	0.49	0.0000811	**<u>0.0057377</u>**
Glucose	Sugar	HMDB0304632	0.463	0.0001912	**<u>0.0104239</u>**
Linoleic acid	PUFA	HMDB0000673	0.444	0.0003404	**<u>0.0156198</u>**
Linolenic acid	PUFA		0.438	0.0004176	**<u>0.0182754</u>**
PC(P-16:0/18:2)	Plasmalogen		-0.434	0.0004603	**<u>0.0197991</u>**
PC(16:0/20:3)	Phosphatidylcholine		-0.394	0.0014442	**<u>0.0457456</u>**
LysoPC(20:3/0:0)	Lysophosphatidylcholine	HMDB0010393	0.391	0.0015912	**<u>0.0489736</u>**
PC(36:5)	Phosphatidylcholine		-0.372	0.0026235	**0.0727152**
Sterol C27H44O2	Sterol		0.367	0.0030126	**0.0808048**
β-Hydroxymyristic acid	Hydroxy fatty acid	HMDB0061656	0.362	0.0033889	**0.0880582**
ACar 5:0 (valeryl/isovalerylcarnitine)	Acylcarnitine		0.36	0.0035870	**0.0928432**
L -Tyrosine	Amino acid	HMDB0000158	0.359	0.0036099	**0.0930739**
DG(16:1/18:1)	Diacylglycerol	HMDB0007130	0.359	0.0036389	**0.0934589**
N,N-Dimethylarginine	Amino acid	HMDB0251395	0.349	0.0046668	0.1120707
Docosahexaenoic acid	PUFA	HMDB0002183	0.341	0.0056707	0.1294487
3-Hydroxybutanoic acid	Hydroxy acid	HMDB0000011	0.339	0.0060017	0.1330786
7alpha-Hydroxy-3-oxo-4-cholestenoic acid	Primary bile acid	HMDB0012458	0.337	0.0062485	0.1362777

**Metabolite**	**Ontology**	**HMDB ID**	**Cohen's *d***	***p* value**	***q* value**
Myristic acid (C14:0)	SAFA	HMDB0000806	0.331	0.0073247	0.1527389
ACar 10:0 (decanoylcarnitine)	Acylcarnitine	HMDB0000651	0.322	0.0090833	0.1765878
LysoPC(20:0)	Lysophosphatidylcholine	HMDB0010390	-0.318	0.0098587	0.1852554
LysoPC(20:4/0:0)	Lysophosphatidylcholine	HMDB0010395	0.318	0.0099409	0.1857718
Sinapyl alcohol	Polyphenol	HMDB0013070	-0.317	0.0100869	0.1863770
ACar 6:0 (hexanoylcarnitine)	Acylcarnitine	HMDB0000756	0.317	0.0102287	0.1863770
PC(18:2/18:2)	Phosphatidylcholine	HMDB0008138	-0.314	0.0107125	0.1890483
ACar 12:1 (dodecenoylcarnitine)	Acylcarnitine		0.313	0.0112082	0.1934264
ACar 8:0 (octanoylcarnitine)	Acylcarnitine	HMDB0000791	0.311	0.0116581	0.1967823
LysoPE(0:0/20:4)	Lysophosphatidylethanolamine	HMDB0011487	0.311	0.0117442	0.1967823
ACar 16:1	Acylcarnitine		0.308	0.0123435	0.2017370
LysoPC(0:0/20:4)	Lysophosphatidylcholine	HMDB0061699	0.307	0.0127696	0.2051772
PC(16:0/20:4)	Phosphatidylcholine	HMDB0007982	0.297	0.0158962	0.2360309
ACar 10:1	Acylcarnitine		0.296	0.0165199	0.2420499
β-Alanine	Amino acid	HMDB0000056	0.293	0.0172275	0.2464450
Palmitic acid (C16:0)	SAFA	HMDB0000220	0.291	0.0183107	0.2558880
Phe-Trp	Dipeptide	HMDB0029006	0.29	0.0187204	0.2599752
PC(O-16:0/18:2)	Plasmalogen		-0.288	0.0192142	0.2641165
LysoPE(20:4)	Lysophosphatidylethanolamine	HMDB0011517	0.288	0.0194542	0.2651172
ACar 14:1 (tetradecenoylcarnitine)	Acylcarnitine		0.275	0.0254491	0.3117635
Arachidonic acid	PUFA	HMDB0001043	0.275	0.0256414	0.3135418
ACar 12:0 (dodecanoylcarnitine)	Acylcarnitine	HMDB0002250	0.274	0.0260368	0.3160528
ACar 7:0	Acylcarnitine	HMDB0013238	0.273	0.0265946	0.3197979
LysoPC(18:3)	Lysophosphatidylcholine		-0.271	0.0273102	0.3219846
PC 40:6e	Plasmalogen		-0.266	0.0304131	
LysoPC(20:1)	Lysophosphatidylcholine	HMDB0010391	-0.265	0.0311968	0.3419520
Eicosapentaenoic acid (C20:5)	PUFA	HMDB0001999	0.265	0.0313769	0.3432878
PC 36:3e	Plasmalogen		-0.263	0.0324886	0.3495091
LysoPE(22:6)	Lysophosphatidylethanolamine		0.259	0.0352485	0.3638739
3-Hydroxydodecanoylcarnitine	Acylcarnitine		0.258	0.0361037	0.3672203
LysoPC(16:1)	Lysophosphatidylcholine	HMDB0010383	0.256	0.0374549	0.3729786
LysoPC(22:6/0:0)	Lysophosphatidylcholine	HMDB0010404	0.255	0.0380408	0.3765012
Unknown C15H20O4			-0.253	0.0393292	0.3824823
LysoPC(0:0/16:1)	Lysophosphatidylcholine	HMDB0010383	0.25	0.0417746	0.3936043
FAHFA 34:2	Fatty acid ester of hydroxy fatty acid		0.25	0.0423285	0.3960185
Anhydroretinol	Sesquiterpenoid	HMDB0062447	0.249	0.0429273	0.3990213

Metabolite	Ontology	HMDB ID	Cohen's *d*	*p* value	*q* value
Erucamide (13-docosenamide)	Fatty amide	HMDB0244507	0.245	0.0463180	
Unknown C15H21NO4			-0.244	0.0468959	0.4171413
LysoPC(16:1/0:0)	Lysophosphatidylcholine	HMDB0010383	0.244	0.0470218	0.4171413
LysoPC(0:0/22:6)	Lysophosphatidylcholine	HMDB0010404	0.243	0.0483028	0.4186676

ESM Table 4. Differential metabolites (p<0.05; q<0.1 in bold, q<0.05 underlined) between **early-onset GDM** and **control groups** in **normal-weight** individuals.

Metabolite	Ontology	HMDB ID	Cohen's *d*	*p* value	*q* value
Glucose	Sugar	HMDB0304632	0.776	0.0000098	**<u>0.0040662</u>**
Cholic acid	Primary bile acid	HMDB0000619	0.502	0.0016677	0.3370898
LysoPC(18:0)	Lysophosphatidylcholine	HMDB0011128	0.485	0.0034798	0.4692053
ACar 6:0 (hexanoylcarnitine)	Acylcarnitine	HMDB0000756	0.491	0.0091089	0.5935273
Docosatetraenoic acid (C22:4)	PUFA	HMDB0002226	0.528	0.0095471	0.5935273
Deoxycholic acid	Secondary bile acid	HMDB0000626	0.414	0.0158435	0.6496478
Unknown PlaSMA ID-114			-0.48	0.0175977	0.6551578
Oleic acid	MUFA	HMDB0000207	0.452	0.0194670	0.6623277
β-Hydroxymyristic acid	Hydroxy fatty acid	HMDB0061656	0.437	0.0226636	0.6725590
Paraxanthine	Alkaloid	HMDB0001860	0.378	0.0253051	0.6775942
Phe-Trp	Dipeptide	HMDB0029006	0.44	0.0285195	0.6986106
ACar 8:0 (octanoylcarnitine)	Acylcarnitine	HMDB0000791	0.421	0.0294697	0.7051532
Prolylhydroxyproline	Dipeptide	HMDB0006695	-0.436	0.0295875	0.7054331
ACar 10:0 (decanoylcarnitine)	Acylcarnitine	HMDB0000651	0.413	0.0324189	0.7077997
Unknown C10H10N2			-0.399	0.0324738	0.7077997
PC 38:4e	Plasmalogen		-0.437	0.0332210	
Arachidonic acid	PUFA	HMDB0001043	0.422	0.0346483	0.7080580
Linolenic acid	PUFA		0.397	0.0369347	0.7080580
PC 40:8	Phosphatidylcholine		-0.392	0.0376538	0.7087480
Myristic acid (C14:0)	SAFA	HMDB0000806	0.382	0.0409717	0.7324201
Phe-Leu/Ile	Dipeptide		0.544	0.0433102	0.7522180
Indoleacetaldehyde	Indole	HMDB0001190	-0.37	0.0495308	

ESM Table 5. Differential metabolites ($p<0.05$; $q<0.1$ in bold, q<0.05 underlined) between **late-onset GDM** and **control groups** in **normal weight** individuals.

Metabolite	Ontology	HMDB ID	Cohen's *d*	*p* value	*q* value
L-Leucine	Amino acid	HMDB0000687	0.644	0.0022088	0.7219173
ACar 10:2	Acylcarnitine		0.55	0.0027925	0.7219173
Phe-Leu/Ile	Dipeptide		0.494	0.0031439	0.7219173
ACar 12:2	Acylcarnitine		0.523	0.0052709	0.7219173
ACar 14:3	Acylcarnitine		0.484	0.0061279	0.7219173
MG(18:1) (monoolein)	Monoacylglycerol	HMDB0011567	0.551	0.0081206	0.7219173
Cyclo(Leu-Pro)	Cyclic peptide	HMDB0034276	0.488	0.0084798	0.7219173
Estriol	Estrogen steroid	HMDB0000153	0.474	0.0109393	0.7219173
ACar 12:1 (dodecenoylcarnitine)	Acylcarnitine		0.472	0.0114437	0.7219173
Dehydroepiandrosterone sulfate (isomer 1)	Steroid sulfate	HMDB0001032	0.469	0.0116360	0.7219173
Dehydroepiandrosterone sulfate (isomer 2)	Steroid sulfate	HMDB0001032	0.459	0.0126760	0.7219173
ACar 16:4	Acylcarnitine		0.421	0.0131822	0.7219173
2-Piperidinone	Piperidine	HMDB0011749	0.423	0.0139144	0.7219173
HETE	Eicosanoid		0.426	0.0163736	0.7219173
Pipecolic acid	Lysine metabolite	HMDB0000070	0.474	0.0169339	0.7219173
Arachidonic acid	PUFA	HMDB0001043	0.477	0.0178150	0.7219173
ACar 6:0 (hexanoylcarnitine)	Acylcarnitine	HMDB0000756	0.508	0.0192818	0.7367871
L-Phenylalanine	Amino acid	HMDB0000159	0.497	0.0197396	0.7367871
PC 40:5e	Plasmalogen		-0.484	0.0233082	
Cotinine	Alkaloid	HMDB0001046	0.319	0.0237990	0.7625336
ACar 14:2	Acylcarnitine		0.394	0.0278132	0.7759518
Phe-Trp	Dipeptide	HMDB0029006	0.439	0.0283126	0.7759518
Docosatetraenoic acid (C22:4)	PUFA	HMDB0002226	0.432	0.0290916	0.7759518
Acetylcarnitine	Acylcarnitine	HMDB0000201	0.425	0.0291171	0.7759518
Cystine	Amino acid	HMDB0000192	-0.452	0.0293724	0.7759518
LysoPC(16:1)	Lysophosphatidylcholine	HMDB0010383	0.386	0.0294495	0.7759518
ACar 10:1	Acylcarnitine		0.452	0.0296157	0.7759518
Hydroxystearic acid	Hydroxy fatty acid	HMDB0062549	-0.438	0.0327924	0.7930569
3-Hydroxydodecanoylcarnitine	Acylcarnitine		0.405	0.0334803	0.7930569
Androgen C19H30O	Androgen		0.381	0.0353796	0.8082363
LysoPA(22:4)	Lysophosphatidic acid	HMDB0114752	0.367	0.0357849	0.8082363
PC 40:6e	Plasmalogen		-0.43	0.0366056	
Unknown C16H24N4O2			-0.416	0.0382071	0.8119922

Metabolite	Ontology	HMDB ID	Cohen's *d*	*p* value	*q* value
LysoPA(20:4)	Lysophosphatidic acid	HMDB0114742	0.393	0.0406918	0.8214132
Sesquiterpenoid C15H22O2	Sesquiterpenoid		-0.435	0.0410080	0.8214132
PC 36:3e	Plasmalogen		-0.44	0.0413430	0.8214132
PC(O-16:0/18:2)	Plasmalogen		-0.442	0.0415687	0.8214132
LysoPC(0:0/16:1)	Lysophosphatidylcholine	HMDB0010383	0.351	0.0449374	0.8242271
ACar 16:1	Acylcarnitine		0.378	0.0449781	0.8242271
ACar 14:0	Acylcarnitine	HMDB0254979	0.39	0.0469432	0.8309161
ACar 14:1 (tetradecenoylcarnitine)	Acylcarnitine		0.372	0.0498557	0.8587404

ESM Table 6. Differential metabolites ($p<0.05$; $q<0.1$ in bold, $q<0.05$ underlined) between **late-onset GDM** and **early-onset GDM groups** in **normal weight** individuals.

Metabolite	Ontology	HMDB ID	Cohen's *d*	*p* value	*q* value
ACar 10:2	Acylcarnitine		0.949	0.0001262	**0.0762981**
Glucose	Sugar	HMDB0304632	-0.698	0.0005197	0.2033743
Cyclo(Leu-Pro)	Cyclic peptide	HMDB0034276	0.651	0.0033938	0.7525219
ACar 16:4	Acylcarnitine		0.607	0.0038232	0.8164947
Indoleacetaldehyde	Indole	HMDB0001190	0.566	0.0060084	
Unknown C10H10N2			0.633	0.0064934	0.9353742
L -Leucine	Amino acid	HMDB0000687	0.534	0.0083242	0.9353742
Cholic acid	Primary bile acid	HMDB0000619	-0.469	0.0137706	0.9353742
5-Aminovaleric acid betaine (5-AVAB)	Betaine	HMDB0240732	0.492	0.0193699	0.9353742
ACar 12:2	Acylcarnitine		0.502	0.0247862	0.9353742
Paracetamol	Pharmaceutical	HMDB0001859	0.422	0.0248385	0.9353742
L -Tryptophan	Amino acid	HMDB0000929	0.511	0.0286752	0.9353742
ACar 11:1	Acylcarnitine		0.503	0.0300433	0.9353742
PC(18:0/22:6)	Phosphatidylcholine	HMDB0008057	0.476	0.0306746	0.9353742
Dehydroepiandrosterone sulfate (isomer 2)	Steroid sulfate	HMDB0001032	0.455	0.0329351	0.9353742
3-Indolepropionic acid	Indole	HMDB0002302	-0.494	0.0341322	0.9353742
Anhydroretinol	Sesquiterpenoid	HMDB0062447	0.541	0.0349145	0.9353742
L -Valine	Amino acid	HMDB0000883	0.441	0.0356213	0.9353742
2-Piperidinone	Piperidine	HMDB0011749	0.398	0.0395202	0.9415992
LysoPE(18:2)	Lysophosphatidylethanolamine		-0.474	0.0419885	0.9546579

Metabolite	Ontology	HMDB ID	Cohen's *d*	*p* value	*q* value
Uric acid	Xanthine	HMDB0000289	0.448	0.0429839	0.9546579
Cotinine	Alkaloid	HMDB0001046	0.326	0.0432150	0.9546579
ACar 14:3	Acylcarnitine		0.41	0.0499609	0.9600077

ESM Table 7. Differential metabolites (p<0.05; q<0.1 in bold, q<0.05 underlined) between **early-onset GDM** and **control groups** in **overweight** individuals.

Metabolite	Ontology	HMDB ID	Cohen's *d*	*p* value	*q* value
Docosatetraenoic acid (C22:4)	PUFA	HMDB0002226	0.614	0.0000073	**<u>0.0016246</u>**
Glucose	Sugar	HMDB0304632	0.593	0.0000081	**<u>0.0016738</u>**
PC(P-16:0/18:2)	Plasmalogen		-0.57	0.0000723	**<u>0.0094342</u>**
Docosapentaenoic acid (C22:5)	PUFA	HMDB0001976	0.498	0.0002563	**<u>0.0207880</u>**
Oleic acid	MUFA	HMDB0000207	0.472	0.0003258	**<u>0.0228148</u>**
Linolenic acid	PUFA		0.459	0.0005112	**<u>0.0290647</u>**
LysoPC(20:4/0:0)	Lysophosphatidylcholine	HMDB0010395	0.46	0.0005502	**<u>0.0299991</u>**
PC(15:0/18:2)	Phosphatidylcholine		-0.445	0.0013751	**<u>0.0491794</u>**
PC(16:0/20:3)	Phosphatidylcholine		-0.44	0.0015778	**0.0529496**
LysoPC(22:6/0:0)	Lysophosphatidylcholine	HMDB0010404	0.418	0.0017202	**0.0544376**
LysoPC(0:0/20:4)	Lysophosphatidylcholine	HMDB0061699	0.413	0.0017383	**0.0545444**
Linoleic acid	PUFA	HMDB0000673	0.434	0.0019200	**0.0577981**
LysoPC(20:3/0:0)	Lysophosphatidylcholine	HMDB0010393	0.411	0.0022449	**0.0654960**
LysoPC(0:0/22:6)	Lysophosphatidylcholine	HMDB0010404	0.404	0.0024958	**0.0706471**
ACar 10:1	Acylcarnitine		0.388	0.0025637	**0.0716555**
L -Tyrosine	Amino acid	HMDB0000158	0.412	0.0027106	**0.0744913**
ACar 6:0 (hexanoylcarnitine)	Acylcarnitine	HMDB0000756	0.407	0.0030938	**0.0823210**
PC(O-16:0/18:2)	Plasmalogen		-0.405	0.0034471	**0.0874378**
PC(18:2/18:2)	Phosphatidylcholine	HMDB0008138	-0.408	0.0035018	**0.0874378**
Docosahexaenoic acid	PUFA	HMDB0002183	0.38	0.0039226	**0.0945414**
ACar 10:0 (decanoylcarnitine)	Acylcarnitine	HMDB0000651	0.404	0.0049521	0.1072109
PC 36:3e	Plasmalogen		-0.383	0.0056274	0.1142321
ACar 8:0 (octanoylcarnitine)	Acylcarnitine	HMDB0000791	0.392	0.0057937	0.1160841
β-Hydroxymyristic acid	Hydroxy fatty acid	HMDB0061656	0.356	0.0059573	0.1175909
7alpha-Hydroxy-3-oxo-4-cholestenoic acid	Primary bile acid	HMDB0012458	0.37	0.0063238	0.1235341
ACar 16:1	Acylcarnitine		0.361	0.0063651	0.1238021
ACar 12:1 (dodecenoylcarnitine)	Acylcarnitine		0.361	0.0068676	0.1297828

**Metabolite**	**Ontology**	**HMDB ID**	**Cohen's *d***	***p* value**	***q* value**
PC 40:6e	Plasmalogen		-0.374	0.0071790	
PC(36:5)	Phosphatidylcholine		-0.36	0.0091290	0.1533489
ACar 14:1 (tetradecenoylcarnitine)	Acylcarnitine		0.347	0.0097433	0.1580784
LysoPC(22:5)	Lysophosphatidylcholine	HMDB0010402	0.352	0.0100002	0.1597382
ACar 14:2	Acylcarnitine		0.334	0.0122799	0.1855039
PE-Cer(d34:1)	Ceramide phosphoethanolamine		-0.351	0.0126253	0.1887277
LysoPE(0:0/20:4)	Lysophosphatidylethanolamine	HMDB0011487	0.325	0.0129734	0.1913761
N,*N*-Dimethylarginine	Amino acid	HMDB0251395	0.335	0.0143881	0.2067167
Sinapyl alcohol	Polyphenol	HMDB0013070	-0.339	0.0153325	0.2147189
3-Hydroxybutanoic acid	Hydroxy acid	HMDB0000011	0.334	0.0159204	0.2197894
PE(16:0/18:2)	Phosphatidylethanolamine	HMDB0008928	-0.34	0.0168692	0.2253292
DG(16:1/18:1)	Diacylglycerol	HMDB0007130	0.322	0.0172673	0.2274492
Arachidonic acid	PUFA	HMDB0001043	0.324	0.0178000	0.2308104
Erucamide (13-docosenamide)	Fatty amide	HMDB0244507	0.332	0.0178844	
Palmitic acid (C16:0)	SAFA	HMDB0000220	0.288	0.0228014	0.2759218
FAHFA 34:2	Fatty acid ester of hydroxy fatty acid		0.293	0.0233389	0.2792271
Eicosapentaenoic acid (C20:5)	PUFA	HMDB0001999	0.308	0.0240851	0.2855865
SM d42:3	Sphingomyelin		-0.328	0.0240901	
3-Hydroxydodecanoylcarnitine	Acylcarnitine		0.319	0.0241851	0.2862617
β-Alanine	Amino acid	HMDB0000056	0.311	0.0244411	0.2882664
PC(17:0/18:2)	Phosphatidylcholine		-0.315	0.0247044	0.2885837
ACar 12:0 (dodecanoylcarnitine)	Acylcarnitine	HMDB0002250	0.323	0.0247764	0.2885837
ACar 16:2	Acylcarnitine		0.291	0.0256222	0.2940019
LysoPC(18:0)	Lysophosphatidylcholine	HMDB0011128	0.274	0.0313455	0.3251344
Androgen C19H30O	Androgen		0.26	0.0337042	0.3364659
PC(16:0/20:4)	Phosphatidylcholine	HMDB0007982	0.291	0.0337376	0.3364659
ACar 5:0 (valeryl/isovalerylcarnitine)	Acylcarnitine		0.286	0.0347993	0.3403204
LysoPC(20:0)	Lysophosphatidylcholine	HMDB0010390	-0.29	0.0352842	0.3431438
PC(18:0/20:4)	Phosphatidylcholine		0.255	0.0362374	0.3473362
LysoPE(22:6)	Lysophosphatidylethanolamine		0.282	0.0368168	0.3492656
Octadecanedioic acid	Dicarboxylic acid	HMDB0000782	0.292	0.0379299	0.3546543
Myristic acid (C14:0)	SAFA	HMDB0000806	0.255	0.0403986	0.3658755
Sterol C27H44O2	Sterol		0.275	0.0406915	0.3663314
ACar 14:3	Acylcarnitine		0.272	0.0412758	0.3678734

Metabolite	Ontology	HMDB ID	Cohen's *d*	*p* value	*q* value
Unknown C15H21NO4			-0.279	0.0446031	0.3798970
PC(32:2)	Phosphatidylcholine		-0.28	0.0460093	0.3864317
LysoPE(20:4)	Lysophosphatidylethanolamine	HMDB0011517	0.272	0.0471186	0.3922814
SM(d18:1/12:0)	Sphingomyelin		-0.285	0.0477226	0.3943488

ESM Table 8. Differential metabolites ($p<0.05$; $q<0.1$ in bold, $q<0.05$ underlined) between **late-onset GDM** and **control groups** in **overweight** individuals.

Metabolite	Ontology	HMDB ID	Cohen's *d*	*p* value	*q* value
Unknown C15H20O4			-0.614	0.0008248	0.1192684
Sterol C27H44O2	Sterol		0.557	0.0023481	0.2091868
Oleic acid	MUFA	HMDB0000207	0.582	0.0031930	0.2413621
Docosatetraenoic acid (C22:4)	PUFA	HMDB0002226	0.53	0.0033924	0.2481022
ACar 5:0 (valeryl/isovalerylcarnitine)	Acylcarnitine		0.506	0.0046686	0.2776778
Linoleic acid	PUFA	HMDB0000673	0.462	0.0066177	0.3354433
Homo-L-arginine	Amino acid	HMDB0000670	0.41	0.0067136	0.3354433
Docosapentaenoic acid (C22:5)	PUFA	HMDB0001976	0.472	0.0086663	0.3753005
gamma-Butyrobetaine	Betaine	HMDB0001161	-0.432	0.0125384	0.4493119
PC(32:1)	Phosphatidylcholine		0.417	0.0132481	0.4525219
L-Threonine	Amino acid	HMDB0000167	0.411	0.0140740	0.4525219
Myristic acid (C14:0)	SAFA	HMDB0000806	0.56	0.0147450	0.4643881
DG(16:1/18:1)	Diacylglycerol	HMDB0007130	0.433	0.0161713	0.4780958
LysoPC(18:3)	Lysophosphatidylcholine		-0.47	0.0166874	0.4883461
Urocanic acid	Histidine metabolite	HMDB0000301	0.244	0.0221942	0.5388180
ACar 7:0	Acylcarnitine	HMDB0013238	0.312	0.0226364	0.5425596
PC(36:5)	Phosphatidylcholine		-0.393	0.0231499	0.5499748
Glycine betaine	Betaine	HMDB0000043	-0.39	0.0263739	0.5714190
LysoPC(20:0)	Lysophosphatidylcholine	HMDB0010390	-0.371	0.0320864	0.5997639
N,*N*-Dimethylarginine	Amino acid	HMDB0251395	0.374	0.0323598	0.6001714
Unknown C16H24N4O2			-0.458	0.0344908	0.6085745
Tetradecanoic acid (C14:0)	Fatty acid	HMDB0000806	0.309	0.0369800	0.6200509
Linolenic acid	PUFA		0.394	0.0411671	0.6366706
3-Hydroxybutanoic acid	Hydroxy acid	HMDB0000011	0.346	0.0427338	0.6456223
LysoPE(20:3)	Lysophosphatidylethanolamine		0.352	0.0441225	0.6484044
MG(18:0)	Monoacylglycerol	HMDB0011131	0.346	0.0494430	0.6698470

ESM Table 9. Differential metabolites (p<0.05; q<0.1 in bold, q<0.05 underlined) between **late-onset GDM** and **early-onset GDM groups** in **overweight** individuals.

Metabolite	Ontology	HMDB ID	Cohen's *d*	*p* value	*q* value
PC(15:0/18:2)	Phosphatidylcholine		0.69	0.0002645	**0.0977443**
L -Threonine	Amino acid	HMDB0000167	0.698	0.0003678	0.1061927
PE(16:0/20:4)	Phosphatidylethanolamine	HMDB0008937	0.665	0.0019955	0.3058172
PE(16:0/18:2)	Phosphatidylethanolamine	HMDB0008928	0.649	0.0024794	0.3058172
LysoPC(22:6/0:0)	Lysophosphatidylcholine	HMDB0010404	-0.495	0.0053713	0.4408313
PC(16:0/18:2)	Phosphatidylcholine		0.515	0.0057791	0.4470049
PC(32:2)	Phosphatidylcholine		0.533	0.0060613	0.4509230
LysoPC(0:0/22:6)	Lysophosphatidylcholine	HMDB0010404	-0.482	0.0062096	0.4509230
Homo- L -arginine	Amino acid	HMDB0000670	0.439	0.0072499	0.4648612
Unknown C15H20O4			-0.609	0.0073260	0.4648612
SM d32:2	Sphingomyelin		0.525	0.0074175	0.4648612
PC(14:0/16:0)	Phosphatidylcholine	HMDB0007869	0.508	0.0074361	0.4648612
1-Methylnicotinamide	Nicotinamide	HMDB0000699	0.388	0.0097931	0.5520628
Unknown C16H24N4O2			-0.491	0.0102660	0.5690780
LysoPC(20:4/0:0)	Lysophosphatidylcholine	HMDB0010395	-0.443	0.0116781	0.6064410
Glucose	Sugar	HMDB0304632	-0.584	0.0122486	0.6140327
PC(33:1)	Phosphatidylcholine		0.459	0.0134437	0.6280063
LysoPC(22:5)	Lysophosphatidylcholine	HMDB0010402	-0.429	0.0139596	0.6280063
PE(16:0/22:6)	Phosphatidylethanolamine		0.503	0.0150646	0.6433207
SM(d18:1/12:0)	Sphingomyelin		0.431	0.0167387	0.6588511
Lenticin (tryptophan betaine)	Betaine	HMDB0061115	-0.446	0.0197688	0.7070013
L -Histidine	Amino acid	HMDB0000177	0.454	0.0199694	0.7075602
Urocanic acid	Histidine metabolite	HMDB0000301	0.257	0.0253271	0.7379471
PE-Cer(d34:1)	Ceramide phosphoethanolamine		0.426	0.0260462	0.7379471
ACar 12:2	Acylcarnitine		-0.405	0.0293286	0.7542145
PC(17:0/18:2)	Phosphatidylcholine		0.409	0.0298194	0.7542145
Tetradecanoic acid (C14:0)	Fatty acid	HMDB0000806	0.38	0.0373580	0.7930149
gamma-Butyrobetaine	Betaine	HMDB0001161	-0.395	0.0388639	0.7930149
PC(32:1)	Phosphatidylcholine		0.347	0.0392365	0.7981691

Metabolite	Ontology	HMDB ID	Cohen's *d*	*p* value	*q* value
SM d33:1	Sphingomyelin		0.382	0.0410316	0.8031786
PE(36:3)	Phosphatidylethanolamine		0.349	0.0447467	0.8120236
LysoPC(0:0/20:4)	Lysophosphatidylcholine	HMDB0061699	-0.358	0.0454108	0.8120236
PC(38:5)	Phosphatidylcholine		-0.396	0.0480518	0.8132551
PE(P-16:0/22:6)	Plasmalogen	HMDB0005780	0.379	0.0495059	0.8132551
Unknown C22H28O2			-0.38	0.0496337	0.8132551

ESM Table 10. Metabolites included in the best-fitting multivariate PLS-DA model to explain the differences between **early-onset GDM** and **control groups** in **normal-weight** individuals.

Metabolite	Ontology	HMDB ID	ID level	MUVR PLS Order	MUVR PLS Rank
Glucose	Sugar	HMDB0304632	2	1	1.25
LysoPC(18:0)	Lysophosphatidylcholine	HMDB0011128	2	2	19.45
Cholic acid	Primary bile acid	HMDB0000619	1	3	20.91
ACar 6:0 (hexanoylcarnitine)	Acylcarnitine	HMDB0000756	2	4	21.43
Docosatetraenoic acid (C22:4)	PUFA	HMDB0002226	2	5	27.87
Phe-Leu/Ile	Dipeptide		2	6	35.14
Oleic acid	MUFA	HMDB0000207	2	7	42.22
LysoPC(16:1)	Lysophosphatidylcholine	HMDB0010383	2	8	45.15
Prolylhydroxyproline	Dipeptide	HMDB0006695	2	9	45.49
Deoxycholic acid	Secondary bile acid	HMDB0000626	2	10	46.48
PC 38:4e	Plasmalogen		3	11	51.46
Pipecolic acid	Lysine metabolite	HMDB0000070	1	12	52.72
β-Hydroxymyristic acid	Hydroxy fatty acid	HMDB0061656	2	13	53.78
ACar 10:0 (decanoylcarnitine)	Acylcarnitine	HMDB0000651	1	14	58.08
ACar 8:0 (octanoylcarnitine)	Acylcarnitine	HMDB0000791	1	15	58.55
Myristic acid (C14:0)	SAFA	HMDB0000806	2	16	58.83
Linolenic acid	PUFA		3	17	63.01
Linoleic acid	PUFA	HMDB0000673	2	18	63.99
ACar 14:1 (tetradecenoylcarnitine)	Acylcarnitine		1	19	64.60
Unknown PlaSMA ID-114			4	20	64.84
Arachidonic acid	PUFA	HMDB0001043	2	21	65.93
Phe-Trp	Dipeptide	HMDB0029006	2	22	66.04

Metabolite	Ontology	HMDB ID	ID level	MUVR PLS Order	MUVR PLS Rank
ACar 12:1 (dodecenoylcarnitine)	Acylcarnitine		1	23	70.70
LysoPC(16:1/0:0)	Lysophosphatidylcholine	HMDB0010383	2	24	75.21
LysoPC(0:0/16:1)	Lysophosphatidylcholine	HMDB0010383	2	25	76.20
ACar 16:1	Acylcarnitine		2	26	76.25
Unknown C10H10N2			4	27	76.45
ACar 12:0 (dodecanoylcarnitine)	Acylcarnitine	HMDB0002250	1	28	77.60
Paraxanthine	Alkaloid	HMDB0001860	1	29	81.62
Docosapentaenoic acid (C22:5)	PUFA	HMDB0001976	2	30	81.74

ESM Table 11. Metabolites included in the best fitting multivariate PLS-DA model to explain the differences between **late-onset GDM** and **control groups** in **normal-weight** individuals.

Metabolite	Ontology	HMDB ID	ID level	MUVR PLS Order	MUVR PLS Rank
L -Leucine	Amino acid	HMDB0000687	1	1	23.19
ACar 12:1 (dodecenoylcarnitine)	Acylcarnitine		1	2	23.95
ACar 14:3	Acylcarnitine		2	3	26.40
ACar 12:2	Acylcarnitine		2	4	29.74
Phe-Leu/Ile	Dipeptide		2	5	31.73
ACar 6:0 (hexanoylcarnitine)	Acylcarnitine	HMDB0000756	2	6	32.86
ACar 14:2	Acylcarnitine		2	7	33.13
ACar 14:1 (tetradecenoylcarnitine)	Acylcarnitine		1	8	33.47
PC 40:6e	Plasmalogen		3	9	37.39
Sesquiterpenoid C15H22O2	Sesquiterpenoid		3	10	38.02
Pipecolic acid	Lysine metabolite	HMDB0000070	1	11	40.70
gamma-Butyrobetaine	Betaine	HMDB0001161	1	12	42.07
PC 40:5e	Plasmalogen		2	13	44.41
ACar 12:0 (dodecanoylcarnitine)	Acylcarnitine	HMDB0002250	1	14	44.75
ACar 10:2	Acylcarnitine		2	15	44.78
ACar 16:2	Acylcarnitine		2	16	50.76
ACar 14:0	Acylcarnitine	HMDB0254979	1	17	50.98
3-Hydroxydodecanoylcarnitine	Acylcarnitine		2	18	54.23
ACar 16:1	Acylcarnitine		2	19	56.09

Metabolite	Ontology	HMDB ID	ID level	MUVR PLS Order	MUVR PLS Rank
ACar 10:1	Acylcarnitine		2	20	56.56
L -Phenylalanine	Amino acid	HMDB0000159	1	21	58.89
MG(18:1) (monoolein)	Monoacylglycerol	HMDB0011567	2	22	60.45
ACar 10:0 (decanoylcarnitine)	Acylcarnitine	HMDB0000651	1	23	64.99
Hydroxystearic acid	Hydroxy fatty acid	HMDB0062549	2	24	70.23
ACar 16:4	Acylcarnitine		2	25	72.44
				26	72.77
HETE	Eicosanoid		3	27	73.40
Estriol	Estrogen steroid	HMDB0000153	2	28	78.92
ACar 8:0 (octanoylcarnitine)	Acylcarnitine	HMDB0000791	1	29	79.83
Cotinine	Alkaloid	HMDB0001046	2	30	79.98
Cyclo(Leu-Pro)	Cyclic peptide	HMDB0034276	2	31	80.92
N-Methyllysine	Amino acid	HMDB0002038	2	32	82.21

ESM Table 12. Metabolites included in the best fitting multivariate PLS-DA model to explain the differences between **early-onset GDM** and **control groups** in **overweight** individuals.

Metabolite	Ontology	HMDB ID	ID level	MUVR PLS Order	MUVR PLS Rank
Glucose	Sugar	HMDB0304632	2	1	3.06
Docosatetraenoic acid (C22:4)	PUFA	HMDB0002226	2	2	9.68
PC(P-16:0/18:2)	Plasmalogen		2	3	12.32
PC 40:6e	Plasmalogen		3	4	32.42
LysoPC(20:4/0:0)	Lysophosphatidylcholine	HMDB0010395	2	5	33.04
L -Tyrosine	Amino acid	HMDB0000158	1	6	34.51
LysoPC(0:0/20:4)	Lysophosphatidylcholine	HMDB0061699	2	7	35.54
Docosapentaenoic acid (C22:5)	PUFA	HMDB0001976	2	8	35.82
ACar 14:1 (tetradecenoylcarnitine)	Acylcarnitine		1	9	36.73
LysoPC(20:3/0:0)	Lysophosphatidylcholine	HMDB0010393	2	10	41.42
ACar 16:1	Acylcarnitine		2	11	42.13
ACar 14:0	Acylcarnitine	HMDB0254979	1	12	42.35
ACar 12:1 (dodecenoylcarnitine)	Acylcarnitine		1	13	43.30
ACar 14:2	Acylcarnitine		2	14	44.74
ACar 12:0 (dodecanoylcarnitine)	Acylcarnitine	HMDB0002250	1	15	45.38

Metabolite	Ontology	HMDB ID	ID level	MUVR PLS Order	MUVR PLS Rank
Oleic acid	MUFA	HMDB0000207	2	16	46.76
ACar 16:2	Acylcarnitine		2	17	49.73
Erucamide (13-docosenamide)	Fatty amide	HMDB0244507	2	18	53.57
ACar 10:0 (decanoylcarnitine)	Acylcarnitine	HMDB0000651	1	19	54.62
LysoPE(0:0/20:4)	Lysophosphatidylethanolamine	HMDB0011487	2	20	59.91
5-Aminovaleric acid betaine (5-AVAB)	Betaine	HMDB0240732	1	21	60.76
3-Hydroxydodecanoylcarnitine	Acylcarnitine		2	22	61.22
Linoleic acid	PUFA	HMDB0000673	2	23	63.73
ACar 6:0 (hexanoylcarnitine)	Acylcarnitine	HMDB0000756	2	24	64.38
ACar 14:3	Acylcarnitine		2	25	65.90
LysoPC(22:6/0:0)	Lysophosphatidylcholine	HMDB0010404	2	26	66.65
β-Alanine	Amino acid	HMDB0000056	2	27	72.64
ACar 8:0 (octanoylcarnitine)	Acylcarnitine	HMDB0000791	1	28	72.72
Linolenic acid	PUFA		3	29	73.00
Acetylcarnitine	Acylcarnitine	HMDB0000201	1	30	74.36
LysoPC(0:0/22:6)	Lysophosphatidylcholine	HMDB0010404	2	31	75.62
ACar 10:1	Acylcarnitine		2	32	76.82

ESM Table 13. Metabolites included in the best fitting multivariate PLS-DA model to explain the differences between **late-onset GDM** and **control groups** in **overweight** individuals.

Metabolite	Ontology	HMDB ID	ID level	MUVR PLS Order	MUVR PLS Rank
Unknown C15H20O4			4	1	7.89
Oleic acid	MUFA	HMDB0000207	2	2	8.02
Docosatetraenoic acid (C22:4)	PUFA	HMDB0002226	2	3	9.22
Myristic acid (C14:0)	SAFA	HMDB0000806	2	4	9.90
Sterol C27H44O2	Sterol		3	5	12.88
gamma-Butyrobetaine	Betaine	HMDB0001161	1	6	17.60
Docosapentaenoic acid (C22:5)	PUFA	HMDB0001976	2	7	19.45
Linoleic acid	PUFA	HMDB0000673	2	8	21.00
Unknown C16H24N4O2			4	9	34.22
Linolenic acid	PUFA		3	10	36.49
ACar 5:0 (valeryl/isovalerylcarnitine)	Acylcarnitine		2	11	42.53
Homo-ʟ-arginine	Amino acid	HMDB0000670	2	12	45.93

Metabolite	Ontology	HMDB ID	ID level	MUVR PLS Order	MUVR PLS Rank
PC(32:1)	Phosphatidylcholine		3	13	50.43
DG(16:1/18:1)	Diacylglycerol	HMDB0007130	2	14	51.11
β-Hydroxymyristic acid	Hydroxy fatty acid	HMDB0061656	2	15	52.48
PE(16:0/20:4)	Phosphatidylethanolamine	HMDB0008937	2	16	54.64
				17	56.07
Glycine betaine	Betaine	HMDB0000043	1	18	60.18
PE(16:0/22:6)	Phosphatidylethanolamine		2	19	69.35
L -Threonine	Amino acid	HMDB0000167	1	20	70.06
Unknown $C_{10}H_{14}N_2O_2$			4	21	76.54
LysoPC(20:0)	Lysophosphatidylcholine	HMDB0010390	2	22	80.31
				23	80.69
PC(36:5)	Phosphatidylcholine		3	24	82.36
Lenticin (tryptophan betaine)	Betaine	HMDB0061115	2	25	86.19
N,*N*-Dimethylarginine	Amino acid	HMDB0251395	2	26	89.80
Urocanic acid	Histidine metabolite	HMDB0000301	1	27	91.41
LysoPC(18:3)	Lysophosphatidylcholine		2	28	91.55
Unknown $C_6H_{13}N_3O_2$			4	29	95.10
LysoPE(20:3)	Lysophosphatidylethanolamine		2	30	96.49
PC(38:5)	Phosphatidylcholine		2	31	98.42
PC(16:0/20:4)	Phosphatidylcholine	HMDB0007982	2	32	98.75
ACar 7:0	Acylcarnitine	HMDB0013238	2	33	98.93
Phe-Trp	Dipeptide	HMDB0029006	2	34	100.28

ESM Table 14. Metabolites included in the best-fitting multivariate PLS-DA model to explain the differences between **late-onset GDM** and **early-onset GDM** in **normal-weight** individuals.

Metabolite	Ontology	HMDB ID	ID level	MUVR PLS Order	MUVR PLS Rank
ACar 10:2	Acylcarnitine		2	1	2.06
Glucose	Sugar	HMDB0304632	2	2	8.23
Unknown $C_{10}H_{14}N_2O_2$			4	3	9.49
ACar 16:4	Acylcarnitine		2	4	11.72
Cyclo(Leu-Pro)	Cyclic peptide	HMDB0034276	2	5	16.80
Unknown $C_{10}H_{10}N_2$			4	6	27.36

Metabolite	Ontology	HMDB ID	ID level	MUVR PLS Order	MUVR PLS Rank
Indoleacetaldehyde	Indole	HMDB0001190	2	7	29.46
L -Leucine	Amino acid	HMDB0000687	1	8	36.76
ACar 12:2	Acylcarnitine		2	9	36.96
ACar 14:3	Acylcarnitine		2	10	38.25
Lenticin (tryptophan betaine)	Betaine	HMDB0061115	2	11	46.60
Anhydroretinol	Sesquiterpenoid	HMDB0062447	2	12	47.02
ACar 11:1	Acylcarnitine		2	13	48.16
L -Tryptophan	Amino acid	HMDB0000929	1	14	53.50
3-Indolepropionic acid	Indole	HMDB0002302	1	15	55.38
Paracetamol	Pharmaceutical	HMDB0001859	2	16	70.65
5-Aminovaleric acid betaine (5-AVAB)	Betaine	HMDB0240732	1	17	71.30
Cholic acid	Primary bile acid	HMDB0000619	1	18	72.97
LysoPE(18:2)	Lysophosphatidylethanolamine		2	19	73.21
Unknown PlaSMA ID-114			4	20	74.05
β-Hydroxymyristic acid	Hydroxy fatty acid	HMDB0061656	2	21	75.72
ACar 14:2	Acylcarnitine		2	22	77.83
ACar 8:0	Acylcarnitine	HMDB0000791	1	23	78.93
Uric acid	Xanthine	HMDB0000289	2	24	79.84
L -Valine	Amino acid	HMDB0000883	2	25	80.96
Palmitic acid (C16:0)	SAFA	HMDB0000220	2	26	81.35
Dehydroepiandrosterone sulfate (isomer 2)	Steroid sulfate	HMDB0001032	2	27	85.11
PC(18:0/22:6)	Phosphatidylcholine	HMDB0008057	2	28	86.36
gamma-Butyrobetaine	Betaine	HMDB0001161	1	29	88.11
PC 40:5e	Plasmalogen		2	30	90.24

ESM Table 15. Metabolites included in the best-fitting multivariate PLS-DA model to explain the differences between **late-onset GDM** and **early-onset GDM groups** in **overweight** individuals.

Metabolite	Ontology	HMDB ID	ID level	MUVR PLS Order	MUVR PLS Rank
Glucose	Sugar	HMDB0304632	2	1	3.37
Unknown C15H20O4			4	2	5.98
PC(15:0/18:2)	Phosphatidylcholine		2	3	9.65
PE(16:0/18:2)	Phosphatidylethanolamine	HMDB0008928	2	4	13.86
L -Threonine	Amino acid	HMDB0000167	1	5	15.49
PE(16:0/20:4)	Phosphatidylethanolamine	HMDB0008937	2	6	15.56

**Metabolite**	**Ontology**	**HMDB ID**	**ID level**	**MUVR PLS Order**	**MUVR PLS Rank**
PC(16:0/18:2)	Phosphatidylcholine		2	7	19.15
				8	21.44
PC(32:2)	Phosphatidylcholine		3	9	28.73
				10	32.09
				11	36.47
PE-Cer(d34:1)	Ceramide phosphoethanolamine		3	12	40.83
PC(33:1)	Phosphatidylcholine		3	13	41.97
Unknown C16H24N4O2			4	14	42.70
PC(17:0/18:2)	Phosphatidylcholine		2	15	46.42
SM d33:1	Sphingomyelin		2	16	49.06
PC(14:0/16:0)	Phosphatidylcholine	HMDB0007869	2	17	49.83
LysoPC(0:0/22:6)	Lysophosphatidylcholine	HMDB0010404	2	18	52.91
PC(38:5)	Phosphatidylcholine		2	19	57.50
Lenticin (tryptophan betaine)	Betaine	HMDB0061115	2	20	58.19
LysoPC(22:6/0:0)	Lysophosphatidylcholine	HMDB0010404	2	21	58.91
Urocanic acid	Histidine metabolite	HMDB0000301	1	22	59.88
SM d32:2	Sphingomyelin		2	23	60.11
PE(36:3)	Phosphatidylethanolamine		3	24	61.26
gamma-Butyrobetaine	Betaine	HMDB0001161	1	25	63.95
SM(d18:1/12:0)	Sphingomyelin		2	26	65.18
LysoPC(22:5)	Lysophosphatidylcholine	HMDB0010402	2	27	67.58
PE(16:0/22:6)	Phosphatidylethanolamine		2	28	70.80
PC(36:5)	Phosphatidylcholine		3	29	70.94
LysoPC(18:3)	Lysophosphatidylcholine		2	30	71.16
L -Tyrosine	Amino acid	HMDB0000158	1	31	73.59
PC 36:3e	Plasmalogen		3	32	77.64
Unknown C22H28O2			4	33	77.75
PC(P-16:0/18:2)	Plasmalogen		2	34	82.04
PE(18:1/20:4)	Phosphatidylethanolamine	HMDB0009036	2	35	91.70

ESM Table 16. Metabolites included in the best fitting multivariate PLS-DA model to explain the differences between the **total GDM** and **control group** in individuals with **normal weight**.

Metabolite	Ontology	HMDB ID	ID level	MUVR PLS Order	MUVR PLS Rank
Glucose	Sugar	HMDB0304632	2	1	6.39
ACar 6:0 (hexanoylcarnitine)	Acylcarnitine	HMDB0000756	2	2	13.35
Pipecolic acid	Lysine metabolite	HMDB0000070	1	3	14.28
Phe-Leu/Ile	Dipeptide		2	4	20.14
ACar 12:1 (dodecenoylcarnitine)	Acylcarnitine		1	5	22.76
ACar 14:1 (tetradecenoylcarnitine)	Acylcarnitine		1	6	26.43
ACar 10:0 (decanoylcarnitine)	Acylcarnitine	HMDB0000651	1	7	29.12
ACar 12:0 (dodecanoylcarnitine)	Acylcarnitine	HMDB0002250	1	8	30.43
ACar 8:0 (octanoylcarnitine)	Acylcarnitine	HMDB0000791	1	9	31.44
Prolylhydroxyproline	Dipeptide	HMDB0006695	2	10	34.72
Sesquiterpenoid C15H22O2	Sesquiterpenoid		3	11	36.00
LysoPC(18:0)	Lysophosphatidylcholine	HMDB0011128	2	12	39.20
ACar 14:2	Acylcarnitine		2	13	41.70
ACar 16:1	Acylcarnitine		2	14	44.07
Docosatetraenoic acid (C22:4)	PUFA	HMDB0002226	2	15	45.06
LysoPC(16:1)	Lysophosphatidylcholine	HMDB0010383	2	16	49.47
L -Glutamine	Amino acid	HMDB0000641	1	17	49.89
ACar 10:1	Acylcarnitine		2	18	49.92
ACar 16:2	Acylcarnitine		2	19	49.94
N-Methyllysine	Amino acid	HMDB0002038	2	20	50.67
ACar 14:0	Acylcarnitine	HMDB0254979	1	21	54.52
Arachidonic acid	PUFA	HMDB0001043	2	22	60.23
3-Hydroxydodecanoylcarnitine	Acylcarnitine		2	23	61.12
ACar 14:3	Acylcarnitine		2	24	62.23
LysoPC(0:0/16:1)	Lysophosphatidylcholine	HMDB0010383	2	25	63.40
Phe-Trp	Dipeptide	HMDB0029006	2	26	65.00
LysoPC(16:1/0:0)	Lysophosphatidylcholine	HMDB0010383	2	27	65.68
Unknown C16H24N4O2			4	28	71.74
PC 38:4e	Plasmalogen		3	29	71.86
PC 40:6e	Plasmalogen		3	30	74.77
MG(18:1) (monoolein)	Monoacylglycerol	HMDB0011567	2	31	79.70
PC 38:6e	Plasmalogen		3	32	80.68

Metabolite	Ontology	HMDB ID	ID level	MUVR PLS Order	MUVR PLS Rank
PC(P-18:0/22:6)	Plasmalogen		3	33	80.88
ACar 12:2	Acylcarnitine		2	34	81.70
PC(P-16:0/18:2)	Plasmalogen		2	35	88.44

ESM Table 17. Metabolites included in the best fitting multivariate PLS-DA model to explain the differences between the **total GDM** and **control group** in **overweight** individuals.

Metabolite	Ontology	HMDB ID	ID level	MUVR PLS Order	MUVR PLS Rank
Docosatetraenoic acid (C22:4)	PUFA	HMDB0002226	2	1	4.15
Oleic acid	MUFA	HMDB0000207	2	2	8.51
Glucose	Sugar	HMDB0304632	2	3	8.93
Docosapentaenoic acid (C22:5)	PUFA	HMDB0001976	2	4	9.48
Linoleic acid	PUFA	HMDB0000673	2	5	15.58
PC(P-16:0/18:2)	Plasmalogen		2	6	20.41
Linolenic acid	PUFA		3	7	24.22
LysoPC(20:3/0:0)	Lysophosphatidylcholine	HMDB0010393	2	8	36.80
L-Tyrosine	Amino acid	HMDB0000158	1	9	46.76
PC(16:0/20:3)	Phosphatidylcholine		2	10	49.24
ACar 16:1	Acylcarnitine		2	11	57.30
ACar 14:1 (tetradecenoylcarnitine)	Acylcarnitine		1	12	59.63
Sterol C27H44O2	Sterol		3	13	61.09
ACar 12:1 (dodecenoylcarnitine)	Acylcarnitine		1	14	65.45
Acetylcarnitine	Acylcarnitine	HMDB0000201	1	15	65.99
β-Alanine	Amino acid	HMDB0000056	2	16	73.42
N,*N*-Dimethylarginine	Amino acid	HMDB0251395	2	17	77.44
ACar 14:0	Acylcarnitine	HMDB0254979	1	18	78.86
Myristic acid (C14:0)	SAFA	HMDB0000806	2	19	81.20
ACar 14:2	Acylcarnitine		2	20	81.92
ACar 16:2	Acylcarnitine		2	21	82.50
